# Retention rates and reasons for non-retention in exercise oncology trials in the post-treatment phase—a systematic review

**DOI:** 10.1007/s11764-024-01569-4

**Published:** 2024-04-03

**Authors:** S. Hu, E. Guinan, D. Mockler, L. O’Neill

**Affiliations:** 1 School of Pharmacy and Pharmaceutical Sciences, Trinity College Dublin, University of Dublin, Dublin 8, Ireland; 2 Trinity St. James’s Cancer Institute, Dublin, Ireland; 3 Discipline of Physiotherapy, School of Medicine, Trinity College Dublin, University of Dublin, Dublin, Ireland; 4 John Stearne Library, Trinity Centre for Health Sciences, St. James’s Hospital, Dublin, Ireland

**Keywords:** Trial retention, Exercise oncology, Cancer survivorship, Retention strategies

## Abstract

**Purpose:**

Retention is a key marker of trial success. Poor retention can induce bias, reduce statistical power and minimise the validity of trials. This review examined retention rates in exercise trials in cancer survivors, reasons for non-retention and retention strategies utilised.

**Methods:**

A systematic review was conducted using a predefined search strategy in EMBASE RCTs, MEDLINE OVID, CINAHL, Web of Science—Core Collection and Cochrane Central Register of Controlled Trials (CENTRAL). The search was conducted on 27/03/2023. Title and abstract screening, full text review and data extraction were completed in duplicate.

**Results:**

Of 17,524 studies identified, 67 trials involving 6093 participants were included. The median overall retention rate immediately post-intervention was 89.85%, range (52.94–100%) and mean 87.36% (standard deviation 9.89%). Trials involving colorectal cancer survivors only had the highest median retention rate (94.61%), followed by breast (92.74%), prostate (86.00%) and haematological cancers (85.49%). Studies involving mixed cancer cohorts had the lowest retention rate (80.18%). The most common retention strategies were wait-list control groups, regular check-ins/reminders and free exercise equipment. Common reasons for non-retention were lost to follow-up, health problems, personal reasons including family/work commitments and travel burden, and disease progression.

**Conclusions:**

Retention rates in exercise oncology trials are approximately 90% immediately post-interventions. Our previous work highlighted variable suboptimal recruitment rates of median 38% (range 0.52–100%). Recruitment rather than retention should be prioritised for methodology research in exercise oncology.

**Implications for cancer survivors:**

Optimising the quality of exercise oncology trials is critical to informing high quality survivorship care.

**PROSPERO registration number:** CRD42023421359.

**Supplementary Information:**

The online version contains supplementary material available at 10.1007/s11764-024-01569-4.

## Background

Exercise prescription is a relatively low-cost intervention which has been shown to positively impact many side effects caused by cancer and its treatments. Robust evidence, including systematic review of randomised clinical trials, asserts that trice weekly moderate to vigorous aerobic exercise and/or twice weekly resistance exercise training has positive impacts on anxiety, depression, cancer-related fatigue, quality-of-life and self-reported physical functioning [1]. Despite this, insufficient evidence exists regarding the role of exercise in cancer in a number of key areas including less commonly studied cancer types, the tumour microenvironment and other sequelae of cancer. Answering these questions will require complex multidisciplinary collaboration[1–3]. However, heterogeneity in recruitment and retention rates observed across trials is a challenge in exercise oncology research, especially amongst less common cancer diagnosis types, thus, potentially diminishing the ability to understand intervention effects and generalizability of findings across cancer types. Consequently, considerable questions remain in exercise oncology, and it is imperative that future trial design is optimised to help provide strong evidence base and guide implementation into clinical care.

Optimising the methodological quality, including the design, conduct, analysis and standard of reporting in trials, is essential to producing relevant, accessible and influential results that can reliably inform evidence-based practice [4]. Regulated and non-regulated clinical trials are expensive and time-consuming to run and successfully deliver and can be compromised by sub-optimal trial recruitment and loss of outcome data [5, 6]. Our previous work in trial methodology in exercise oncology examining trial recruitment rates highlighted reported recruitment rates of 38% [5]. Looking beyond recruitment, the ability to retain participants in these trials is also likely a challenge. Retention is a key marker of success of trials that needs to be considered with recruitment. Poor retention can induce bias, reduce statistical power and minimise the validity of trials [6, 7]. Retention in exercise oncology trials has been underexplored, but it is well acknowledged to be challenging. A systematic review in advanced cancer only found an attrition rate of 25% to be typical [8]. Power calculations in exercise trials typically estimate a non-retention rate of at least 20% [9]; however, as this figure is not based on systematically reviewed data, it may not accurately reflect trials in this field results in under- or over resourcing of trials accrual targets. Reasons for poor retention in such trials are likely to be multifactorial and require investigation. Therefore, this review, which provides the first standardised data of this type, has the potential to positively inform accurate accrual targets and retention strategies in exercise oncology clinical trials.

There remains an opportunity to develop our understanding of trial retention strategies in this field and map specific strategies on the reasons for non-retention observed in these trials. Retention is one of the top three research priorities for the UK clinical trial community. There is a need to address the gap in evidence-based approaches to improving participant retention in randomised trials. The Prioritising Retention in Randomised Trials (PRioRiTY) II project was a priority setting partnership (PSP) which aimed to identify and prioritise the top ten unanswered questions and uncertainties in trial retention [10]. The PRioRiTy II PSP found that the key stakeholders involved in randomised trials such as staff, researchers and patients/public believe future research on improvements to retention should focus primarily on individual motivation to complete trials, how trials can better use routine clinical care and existing data collection pathways and how burden to participants can be minimised through trial design [10]. The top ten questions posed should be used to inform future methodology clinical trial research design, and this can be achieved through rigorous testing of retention strategies by embedding studies within a trial (SWATs): self-contained studies that are embedded within a host trial to explore and/or evaluate alternative methods of trial delivery or processes [11].

To this end, we present the results of a systematic review summarising retention rates of randomised controlled trials of exercise in cancer survivorship and capture reasons for non-retention. Our findings may be used as a benchmark of retention rates for researchers in this field to aid future calculation of sample sizes. Information regarding reason for non-retention in such trials will allow for the development/implementation of strategies at the trial planning stage to maximise retention.

## Methods

### Search strategy

A systematic approach based on the PRISMA guidelines was applied in the reporting of this review. A search strategy generated by the subject librarian (DM) was used (Supplementary Material 1) to search EMBASE RCTs, MEDLINE OVID, CINAHL, Web of Science—Core Collection and Cochrane Central Register of Controlled Trials (CENTRAL) on 27/03/2023.

### Eligibility criteria

Inclusion criteria were: (i) involved a population of adult cancer survivors, i.e. adult cancer patients with a histologically confirmed diagnosis of cancer of any type, who had completed primary treatment, e.g. surgery, neoadjuvant/adjuvant chemotherapy and/or radiotherapy; (ii) included a prescribed exercise intervention; (iii) employed a randomised controlled study design; and (iv) reported information regarding retention rates. Exclusion criteria were: (i) articles unavailable as full text; (ii) full text unavailable in English; (iii) cohort studies, systematic reviews, meta-analysis, case studies, letters to the editor and conference proceedings; (iv) secondary papers; (v) including participants under 18 years of age; and (vi) studies wherein retention rate is not provided nor calculable; and (vii) no prescribed exercise interventions or physical activity recommendations or exercise interventions combined with other non-exercise interventions (e.g. nutrition, behavioural interventions etc.).

Exercise was defined as ‘planned, structured and repetitive bodily movement, the objective of which is to improve or maintain physical fitness’. This definition is inclusive of various exercise interventions including aerobic and resistance training, flexibility training such as yoga and general physical activity programmes which followed a defined exercise prescription based on the F.I.T.T. (frequency, intensity, type and time) principle. We used the definition of retention as described by the Standard Protocol Items: Recommendations for Interventional Trials’ (SPIRIT) guidelines. SPIRIT defines non-retention as ‘instances where participants are prematurely “off-study” (i.e. consent withdrawn or lost to follow-up) and thus outcome data cannot be obtained from them’.

### Data extraction

Data extraction was performed using Covidence, an electronic systematic review management system. Title and abstract screening was completed by two independent reviewers (EG and SH). Articles not meeting the predefined inclusion criteria were excluded. Conflicts were resolved by a third independent reviewer (LON). The same process was applied to the full-text review. Details pertaining to author, year of publication, country, participant characteristics, exercise intervention (and control), duration of intervention/trial participation, number and timing of study assessments, retention rates, reasons for non-retention and retention strategies were extracted by SH, where within text information regarding retention was insufficient, retention rates were calculated from information provided in the CONSORT flow diagram presented in the paper. Ten percent of studies were extracted in duplicate independently by EG. Data was compared, and inconsistencies were resolved. Reasons for non-retention were recorded as reported in the individual papers. From these reasons, common themes were identified, and reasons were categorised for ease of reporting. For example, reasons falling under the theme of health such as injuries (unrelated to the study), accidents (unrelated to the study) and psychological distress were grouped together. Any changes deemed necessary to ensure the clarity of results were reviewed and approved by EG and SH. For trials where there was a supervised and then an unsupervised phase of intervention, retention rate at the end of the supervised portion was recorded as end of intervention retention, and retention at the end of the unsupervised intervention was recorded as a follow-up rather than immediate post-intervention.

Following extraction of retention rates, data was analysed, and mean, standard deviation, median and range were calculated. Median (range) data is primarily reported. Retention rates at subsequent follow-ups were organised by timing of follow-up from baseline rather than time from end of intervention, as intervention duration varied. Reasons for non-retention and recruitment strategies were categorised through consensus. Frequency of reasons for non-retention was calculated as a proportion of the total number prematurely off-study to determine the reasons which pose the most serious threat to retention and retention strategies which could help to minimise their incidence. Results are presented by cancer types to enable trialists working with specific tumour types to obtain specific retention data to inform trial planning.

### Risk of bias assessment 

Assessment of risk of bias was not applicable to this systematic review as studies included were neither randomised nor blinded for the outcomes related to trial retention.

## Results

**Fig. 1:**
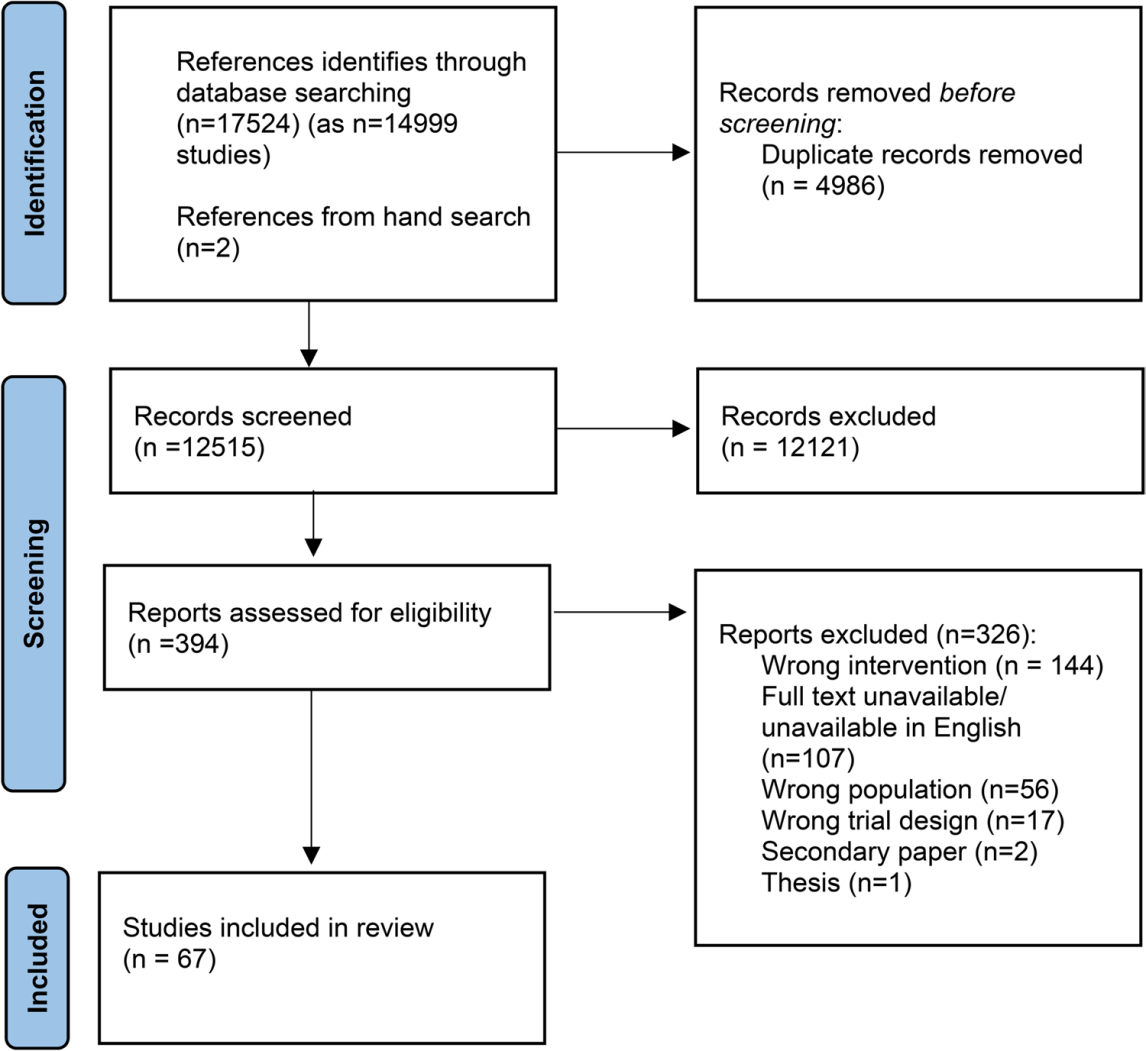
PRISMA flow diagram

The literature search results are presented in the PRISMA flow diagram as Fig. 1. A total of 17,500 papers were identified by the predefined search strategy conducted on March 27th, 2023. Following removal of duplicates, 12,514 studies underwent title and abstract screening. Where more than one paper written on the same study was identified as meeting inclusion criteria, papers were merged using Covidence and the paper with the most comprehensive information regarding retention was included. A total of 381 studies underwent full-text screening, of which 67 studies involving a total of 6093 participants were included in the final review. Manual citation searching of recent comprehensive reviews in the field was performed by SH which yielded two further papers for inclusion. The full list of included studies and their demographic details and post-intervention retention rates can be found in Supplementary Material 2. Trials were published between 2003 and 2023. Most trials were conducted in North America (*n* = 30, 44.78%), with the remainder from Europe (*n* = 19, 28.36%), Australia and New Zealand (*n* = 10, 14.93%), UK (*n* = 3, 4.48%), Asia (*n* = 2, 2.99%) and South America (*n* = 2, 2.99%).

Breast cancer survivors were the most studied cohort, being the most prominent cancer type in 46 trials (68.66%), 34 (50.75%) including breast cancer only and seven (10.45%) where breast cancer accounted for over 50% of participants. This was followed by prostate cancer (*n* = 9, 13.43%), colorectal (*n* = 2, 2.99%), haematological (*n* = 2, 2.99%), colon (*n* = 1, 1.49%), endometrial (*n* = 1, 1.49%), glioma (*n* = 1, 1.49%), head and neck (*n* = 1, 1.49%), lung (*n* = 1, 1.49%), oesophageal (*n* = 1, 1.49%), oesophagogastric (*n* = 1, 1.49%) and ovarian (*n* = 1, 1.49%). Twelve (17.91%) trials included a mix of cancer types, seven of which included a majority of patients with breast cancer.

### Intervention characteristics

Types of interventions prescribed in the included trials are described in Supplementary Material 2. A combination of both aerobic and resistance training was prescribed in the majority of trials (*n* = 27, 40.30%), followed by aerobic exercise only (*n* = 18, 26.87%) and resistance training only (*n* = 17, 25.37%). Other interventions included Physical Activity, Qigong, Circuit Training, Yoga, Pilates, Gymnastics, Relaxation Training, Calisthenics Training, Nordic Walking and Greek Dancing.

### Retention rates of exercise oncology trials: immediate post-intervention

Retention rates immediately post-intervention for individual trials are presented in Supplementary Material 2. Overall retention rate as well as retention rate for intervention and control groups respectively was recorded. The median overall retention rate immediately post-intervention was 89.85%, range (52.94–100%) and mean 87.36% (standard deviation (SD) 9.89%).

When categorised by cancer type (Table 1), trials involving only colorectal cancer survivors had the highest median retention rate of 94.61%, followed by breast (92.74%), prostate (86.00%) and haematological (85.49%). Studies involving mixed cancer cohorts had the lowest median retention rate (80.18%).

**Table 1:** Overall retention rates immediately post intervention

Cancer type	Number of trials	Retention rate (%) Median (range)	Retention rate (%) Mean (standard deviation)
Breast	34	92.74 (52.94–100)	89.57 (10.20)
Prostate	9	86.00 (71.19–100)	83.40 (11.54)
Haematological	2	85.49 (84.09–86.89)	85.49 (1.98)
Colorectal	2	94.61 (93.48–95.74)	94.61 (1.60)
Colon	1	97.44*	-
Endometrial	1	95.00*	-
Glioma	1	94.12*	-
Head & Neck	1	92.31*	-
Lung	1	75.64*	-
Oesophageal	1	91.67*	-
Oesophagogastric	1	93.02*	-
Ovarian	1	78.47*	-
Mixed cancer cohort	12	80.18 (74.36–100)	82.90 (7.75)

^*^Retention rate calculated from *n* = 1 study

### Retention rates of exercise oncology trials: follow-up assessments

Retention rates at subsequent follow-up assessments are presented in Supplementary Material 3, organised by cancer type and timing of follow-up as time from baseline. Again, overall retention rate as well as group-specific retention rates were recorded. The median overall retention rate at follow up was 84.36%, range 96.55–56% and mean 79.56% (SD 13.45%). Unsurprisingly, when organised by timing of follow-up, the further the follow-up from baseline, the lower the retention rate. Three trials included a follow-up assessment where only the intervention group was followed-up, and therefore overall retention rates for these trials were not included in the calculations above [12–14].

Twenty-four studies included follow-up assessments after the post-intervention assessments. These interventions ranged from a minimum of 8 weeks [15–17] to a maximum of 6 months [18, 19] in duration. The median retention rates in studies which included a follow-up assessment within 6 months of the baseline assessment were 88.37% (range 56–98.28%). Unsurprisingly, retention rates reduced at follow-up assessments which were further from baseline. Median retention rates across nine trials completing follow-up assessments at 7–12 months post-baseline were 75.64% (range 46.97–92.31%) [16, 19–25] and 74.04% (range 69.23–78.85%) in one study completing two follow-up assessments at 13–18 months post baseline [20]. Finally, the single study, which included a follow-up at 24 months from baseline, reported a retention rate of 70.59% at this timepoint [18].

### Reasons for trial non-retention

Reasons for non-retention in exercise trials in cancer survivors are outlined in Table 2. A total of 754 participants went off-study immediately post-intervention in the 67 trials. Three trials (3.66%) failed to report specific reasons for non-retention. The most common reasons for non-retention in terms of numbers of participants (*n*) were lost to follow-up (including withdrew/refused/withdrew consent) (*n* = 195, 25.86%), health problems (*n* = 137, 18.17%) and personal reasons (including family/work commitments and travel burden) (*n* = 121, 16.05%). Other reasons are reported in Table 2. Trial-related reasons included unhappy with group allocation, intervention too intense and exercise not meeting expectations.

**Table 2:** Reasons for non-retention during intervention

	Frequency (*n*, %)*
**Reason for non-retention during the intervention**	
Lost to follow-up/not reported	195 (25.86%)
Health problems	137 (18.17%)
Personal reasons including family/work commitments and travel burden	121 (16.05%)
Disease progression	72 (9.55%)
Time constraints	70 (9.28%)
Other reasons (not specified)	52 (6.90%)
No longer eligible	41 (5.44%)
Too much burden	16 (2.12%)
Deceased	15 (1.99%)
Trial-related reasons	13 (1.72%)
Missing end-point data	13 (1.72%)
COVID-19 pandemic	5 (0.66%)
Recruitment failure	4 (0.53%)
**Reason for non-retention at subsequent follow-up assessments**	
Lost to follow-up	65 (43.62%)
Health reasons	29 (19.46%)
Time constraints	15 (10.07%)
Other reasons	13 (8.72%)
Personal reasons including work/family commitments	13 (8.72%)
Disease progression	10 (6.71%)
Breast reconstruction surgery	2 (1.34%)
Trial related reasons	1 (0.67%)
Deceased	1 (0.67%)

^*^Frequency refers to the number of participants within the trials

Health problems included injuries (unrelated to the study), accidents (unrelated to the study) and psychological distress. Disease progression included disease recurrence, development of a new malignancy and withdrawal from the control group because treatment was indicated. Lost to follow-up included those who withdrew/refused and withdrew consent. Missing end-point data included one participant whose questionnaire was lost [26]. Recruitment failure occurred in one trials where consent was obtained after randomisation [27]. Non-retention due to the COVID-19 pandemic included two due to lack of sufficient space to perform the exercise intervention at home [28], one due to increased care-giving duties and one due to increased work commitments as a health-care worker and one due to the COVID-19 closure [29].

### Trial retention strategies

Forty-one studies (61.19%) reported retention strategies which are summarised in Table 3.

**Table 3:** Retention strategies

Retention strategy	Number of trials (%)
Wait-list control group	20 (48.78)
Regular check ins/reminders	16 (39.02)
Free exercise equipment	10 (24.39)
Choice of exercise type	8 (19.51)
Free gym membership	5 (12.20)
Choice of time of group exercise	4 (9.76)
Monetary incentive	3 (7.32)
Non-monetary incentives	1 (2.44)
Exercise advice for control group	1 (2.44)

## Discussion

The aim of this work was to review the retention rates of randomised controlled trials in exercise oncology and characterise reasons for non-retention. These findings may assist trialists in determining sample size required to meet statistical power goals, taking into account likely non-retention rates. Furthermore, identification of the most common reasons for non-retention will allow the implementation of appropriate retention strategies to maximise retention. The main finding from this review is a median overall retention rate in exercise oncology trials immediately post-intervention of 89.85%. In contrast, we previously described a recruitment rate of only 38% to post-treatment exercise oncology trials [5]. The high retention rates reported in this review demonstrate that although recruitment may be challenging, once cancer survivors enrol in exercise trials, they are likely to remain engaged notwithstanding slight variance across cancer types. This overall retention rate is comparable to exercise trials following haematopoietic stem cell transplantation (retention rate 88%; *n* = 20 trials) and multimorbidity (retention rate 90%; *n* = 23 trials) [30]. Unsurprisingly, retention rates in this review involving patients with non-metastatic disease, in the post-treatment phase, are higher than those observed in advanced cancer (76%; *n* = 18 studies) [8] and during active chemotherapy treatment where retention rates ranged from 50 to 100% [31]. In the current review, only 20 of the 67 trials reviewed reported non-retention due to disease recurrence or progression: seven trials (36.84%) involving breast cancer survivors, four (21.05%) involving a mixed cancer cohort, two (15.79%) involving prostate cancer survivors, two (10.53%) involving colorectal cancer survivors and one (5.26%) each involving haematological, oesophagogastric, glioma and oesophageal cancer survivors respectively.

The observed rate of almost 90% is high; however, some heterogeneity in retention rates was observed. Almost 50% of trials reviewed (*n* = 33) reported retention rates > 90%, a further 18 reported retention rates between 80 and 90% and only two trials reported retention rates of < 70%. Heterogeneity may be due to many factors including the nature of the intervention under examination (exercise), potential confounders with regard to retention including but not limited to cancer type, time since treatment completion and the principles of exercise prescription applied of the setting of the programme. Given the large number of potential confounders it is not possible to draw conclusions regarding the characteristics of studies that may support retention but future work in this area is warranted.

The primary reason for non-retention from trials reviewed was lost to follow-up, health problems, personal reasons including family/work commitments and travel burden, and disease progression. Encouragingly, over 60% of trials reviewed reported implementing a planned retention strategy to optimise retention; however, this could be improved. Importantly, category A evidence exists for the use of electronic reminders as effective retention strategy in trials [6]. Regular check-in’s/reminders were the second most frequently utilised retention strategy in the trials reviewed which may be a factor for the high retention rates observed. In order to make recommendations regarding these strategies, their efficacy requires investigation, ideally by embedding a Study Within A Trial. We encourage trialists in the field of exercise oncology to consider embedding methodology work to examine these strategies in the future.

In this review we highlight numerous shortcomings in trial reporting impacting the clarity of retention and non-retention data. Issues included situations where there was an imbalance between the total number of who were not retained on trial and the cumulative reasons provided for non-retention within a trial, reasons for non-retention not reported or explicitly described, reasons for non-retention not reported at follow-up assessments, not describing when participants went prematurely off-study (i.e. during the intervention, at post-intervention assessment or at follow-up assessment), absence of CONSORT diagrams and distinguishing from which arm of the trial participants withdrew. Understanding why participants go prematurely off-study, from which arm of the trial and at what timepoint is critical for understanding what retention strategies may be embedded into trials to improve retention and subsequent trial quality. This lack of attention to detail in trial reporting has been previously highlighted in exercise oncology and cancer rehabilitation literature. Meneses-Echavez and colleagues (2019) analysed reporting completeness in 131 RCTs of exercise oncology trials against the TIDieR (template for intervention description and replication) tool and revealed complete reporting ranging from 42 to 96%, with no trial reporting all TIDieR items [32]. Specifically, issues around protocol tailoring, modifications and fidelity were only reported in approximately 50% of trials reviewed, while the justification for the trial and planned protocol were far more frequently reported. Similarly, a review of attention to the principles of exercise training, and reporting of these principles, in exercise trials in breast cancer survivors, found that none of the 67 trials reviewed reported all principles of exercise training with the principles of reversibility and diminishing returns, which both relate to follow-up assessments, only reported in 3% and 22% of trials respectively [33].

Trials prescribing exercise or other behavioural interventions are non-regulated trials and are not legally required to comply with the International Conference of Harmonisation Good Clinical Practice (ICH GCP), which provides a standard for the conduct of clinical trials [34] or with legislation such as the EU Clinical Trial Regulation EU#536/2014 or the EU Medical Device Regulation 2017/745 [35] which apply to regulated trials involving investigational medicinal products or medical devices. Consequently, strategies such as trial monitoring, which ensure best practice, participant safety, integrity, internal validity and overall quality assurance of clinical trials are not routinely embedded in exercise oncology. Adverse events associated with exercise in cancer care are underreported, and consequently the impact of adverse events on trial retention is understudied. Efforts to encourage better monitoring and reporting of adverse events are ongoing and draw on the principles of pharmacological clinical trial reporting. The Exercise Harms Reporting Method (ExHaRM) is a newly published protocol which provides a four-step standardised approach involving: (1) monitoring and identifying adverse outcomes; (2) assessing and recording adverse outcomes; (3) review by harms panel and potential revision of causality; and (4) analyse and reporting [36]. Similarly, frameworks for reporting aerobic and resistance exercise dose, modifications and adherence are now available in exercise oncology [37, 38]. Implementing these frameworks and considering the inclusion of bespoke trial monitoring strategies [39], within the non-regulated setting of exercise oncology trials, would benefit the standard of reporting and quality of trials being conducted in this field.

## Limitations of review

Our research builds on comprehensive work previously published examining recruitment strategies in exercise oncology. While significant efforts have been made by the team to capture all relevant trials, including updated electronic database searching and manual searching of recent reviews, due to the large volume of titles retrieved in the original search, it is possible that some trials may have been missed. Furthermore, as discussed above, sub-optimal trial reporting limited the standardised reporting of retention data for some publications, and consensus discussions were required amongst the research team to agree on the best approach to data extraction. Finally, this review did not include single-arm trials which would have contained valuable insights into retention and if paired with process evaluations may have provided some context to understand the challenges of trial participation for participants.

## Conclusion

From this review we build on our previous report describing recruitment rates to exercise oncology trials [5], to provide trialists working in exercise oncology and cancer rehabilitation with trial retention data across multiple cancer types to inform future trial planning, sample size calculations and implementation strategies in cancer survivorship. Trialists should consider the inclusion of retention strategies to optimise retention and overcome the major reasons for non-retention such as travel for exercise classes. Sub-optimal reporting practices, particularly in relation to how trials were actually implemented and delivered, need to be addressed to advance trial quality in the field. Established frameworks should be used to guide trial reporting and the inclusion of trial monitoring strategies considered to support this. At minimum, retention data should be reported for each arm of the trial and at each assessment timepoint individually, consort diagrams should be included and sub-analysis of reasons for non-retention should add up to the total number reported.

## Supplementary Information

Below is the link to the electronic supplementary material.

Supplementary file1 (DOCX 22 KB)

Supplementary file2 (DOCX 131 KB)

Supplementary file3 (DOCX 53 KB)

## Data Availability

All data generated or analysed during this study are included in this article and its supplementary information files.
