# Supplementary material for: Retention rates and reasons for non-retention in exercise oncology trials in the post-treatment phase—a systematic review
Source: J Cancer Surviv. 2024 Apr 3;19(5):1535–43. doi: 10.1007/s11764-024-01569-4 (PMC12460456; doi:10.1007/s11764-024-01569-4)
Supplement: Supplementary file 1 — Supplementary file1 (DOCX 22 KB) [file 11764_2024_1569_MOESM1_ESM.docx]

**Supplemental Material 1 - Search Strategy**

*Title:* *:* Retention Rates and Reasons for Non-Retention in Exercise Oncology Trials in the Post-Treatment Phase- A Systematic Review

*Authors*: Sofia Hu^1^, David Mockler^4^, Emer Guinan^2,3^, Linda O’Neill^2,3^

*Affiliations*:

1. School of Pharmacy and Pharmaceutical Sciences, Trinity College Dublin, University of Dublin, Dublin, Ireland
2. Trinity St. James’s Cancer Institute, Dublin, Ireland.
3. Discipline of Physiotherapy, School of Medicine, Trinity College Dublin, University of Dublin, Dublin, Ireland.
4. John Stearne Library, Trinity Centre for Health Sciences, St. James’s Hospital, Dublin, Ireland.

*Corresponding Author:* Dr Linda O’Neill, Research Fellow, Trinity St James’s Cancer Institute, Dublin 8, Ireland. Email: loneill4@tcd.ie

Searches run 27/03/23

Number of articles from all searches = 17524

After de duplication = 12539

| Database | Search Strategy |
| --- | --- |
| EMBASE RCTs [3505] | 'neoplasm'/exp OR 'cancer survivor'/exp OR 'childhood cancer survivor'/exp OR 'cancer rehabilitation'/exp OR 'cancer patient'/exp OR 'cancer survival'/exp  (cancer* or neoplas* or tumor* or tumour* or malignan* or carcinoma* or metasta* or oncolog* or leukemi* or leukaemi* or lymphoma* or myeloma* or sarcoma*):ti,ab,kw #1 OR #2  'patient selection'/exp OR 'patient compliance'/exp OR 'patient attitude'/exp  ((recruit* OR enrol* OR retention OR retain* OR adhere* OR compliance) NEAR/5 (strateg*  OR plan* OR program* OR barrier* OR facilitator* OR rate OR rate$)):ti,ab,kw  ((recruit* OR enrol* OR retention OR retain*) NEAR/5 (survivor* OR patient* OR volunteer* OR participant*)):ti,ab,kw  ((Patient* OR criteria OR subject* OR volunteer* OR treatment* OR participant*) NEAR/2 selection*):ti,ab,kw  ((patient* OR volunteer* OR participant*) NEAR/3 (compliance OR dropout* OR 'drop out'  OR engag* OR participat* OR refusal)):ti,ab,kw  #4 OR #5 OR #6 OR #7 OR #8  'exercise'/exp OR 'kinesiotherapy'/exp OR 'physical activity'/exp OR 'physical activity, capacity and performance'/de OR 'training'/de OR 'endurance'/de OR 'exercise tolerance'/de OR 'physical capacity'/de OR 'sport'/exp  (strength* or isometric* or isotonic* or isokinetic* OR exercis*):ti,ab,kw  (resistance NEAR/3 train*):ti,ab,kw  ((physical* or motion* or cardiopulmonary or cardiorespiratory) NEAR/3 (fit* or therap*)):ti,ab,kw  (treadmill* or cross-train* or rowing or sport* OR exercise* OR ‘physical activit*’ OR aerobic* OR run or jog* or running OR walk or walks or walking OR gym* OR yoga oR pilates OR ‘recreation* activit*’ OR zumba or salsa* OR cycling or bicycle or bike or swim* or dance or dancer* or dances or dancing):ti,ab,kw (circuit* NEAR/1 train*):ti,ab,kw  (keep* NEAR/1 (active or fit)):ti,ab,kw  #10 OR #11 OR #12 OR #13 OR #14 OR #15 OR #16  'clinical trial'/de OR 'randomized controlled trial'/de OR 'randomization'/de OR 'single blind procedure'/de OR 'double blind procedure'/de OR 'crossover procedure'/de OR 'placebo'/de OR 'prospective study'/de OR ('randomi?ed controlled' NEXT/1 trial*) OR rct OR 'randomly allocated' OR 'allocated randomly' OR 'random allocation' OR (allocated NEAR/2 random) OR (single NEXT/1 blind*) OR (double NEXT/1 blind*) OR ((treble OR triple) NEAR/1 blind*)  OR placebo*  #3 AND #9 AND #17 AND #18  (Letter OR editorial OR 'conference abstract' OR 'conference review'):it #19 NOT #20 |
| Medline OVID [1825] | Randomized controlled trials as Topic/  Randomized controlled trial/  Random allocation/  Double blind method/  Single blind method/  Clinical trial/  exp Clinical Trials as Topic/ or/1-7  (clinic$ adj trial$1).tw.  ((singl$ or doubl$ or treb$ or tripl$) adj (blind$3 or mask$3)).tw. Placebos/  Placebo$.tw.  Randomly allocated.tw. (allocated adj2 random).tw. or/9-14  8 or 15  Case report.tw.  Letter/  Historical article/  Review of reported cases.pt.  Review, multicase.pt. or/17-21 16 not 22  exp Neoplasms/ OR Cancer Survivors/  (cancer* or neoplas* or tumor* or tumour* or malignan* or carcinoma* or metasta* or oncolog* or leukemi* or leukaemi* or lymphoma* or myeloma* or sarcoma*).ti,ab. or/24-25  "Treatment Adherence and Compliance"/ OR Patient Selection/ OR Patient Compliance/ OR  Patient Dropouts/ OR Patient Participation/ OR exp Patient Satisfaction/  ((recruit* OR enrol* OR retention OR retain* OR adhere* OR compliance) adj5 (strateg* OR plan* OR program* OR barrier* OR facilitator* OR rate OR rate$)).ti,ab.  ((recruit* OR enrol* OR retention OR retain*) adj5 (survivor* OR patient* OR volunteer* OR participant*)).ti,ab.  ((Patient* OR criteria OR subject* OR volunteer* OR treatment* OR participant*) adj2 selection*).ti,ab.  ((patient* OR volunteer* OR participant*) adj3 (compliance OR dropout* OR 'drop out*' OR engag* OR participat* OR refusal)).ti,ab. or/27-31 exp Exercise/ OR exp Exercise Therapy/ OR exp Physical Fitness/ OR exp "physical education and training"/ OR exp "Exercise Movement Techniques"/ or physical endurance/ or exercise tolerance/ OR Physical Exertion/ or exp Sports/ or Dancing/ (strength* or isometric* or isotonic* or isokinetic* or exercis*).ti,ab.  (resistance adj3 train*).ti,ab.  ((physical* or motion* or cardiopulmonary or cardiorespiratory) adj3 (fit* or therap* or activit*)).ti,ab.  (treadmill* or cross-train* or rowing or sport* or exercise* or physical* activit* or aerobic* or run or jog* or running or walk or walks or walking or gym* or yoga or pilates or "recreation* activit*" or zumba or salsa* or cycling or bicycle or bike or swim* or dance or dancer* or dances or dancing or physiotherapy* or physical therap*).ti,ab.  (circuit* adj1 train*).ti,ab. (keep* adj1 (active or fit)).ti,ab.  or/33-39  23 AND 26 AND 32 AND 40 |
| CINAHL [1449] | (MH "Cancer Patients") OR (MH "Cancer Survivors") OR (MH "Neoplasms+") OR (MH  "Rehabilitation, Cancer")  TI (cancer* or neoplas* or tumor* or tumour* or malignan* or carcinoma* or metasta* or oncolog* or leukemi* or leukaemi* or lymphoma* or myeloma* or sarcoma*) OR AB (cancer* or neoplas* or tumor* or tumour* or malignan* or carcinoma* or metasta* or oncolog* or leukemi* or leukaemi* or lymphoma* or myeloma* or sarcoma*) S1 OR S2  (MH "Patient Selection") OR (MH "Patient Compliance") OR (MH "Patient Dropouts") OR  (MH "Patient Attitudes") OR (MH "Patient Satisfaction")  TI ((recruit* OR enrol* OR retention OR retain* OR adhere* OR compliance) N4 (strateg* OR plan* OR program* OR barrier* OR facilitator* OR rate OR rate*)) OR AB ((recruit* OR enrol* OR retention OR retain* OR adhere* OR compliance) N4 (strateg* OR plan* OR program* OR barrier* OR facilitator* OR rate OR rate*))  TI ((recruit* OR enrol* OR retention OR retain*) N4 (survivor* OR patient* OR volunteer* OR participant*)) OR AB ((recruit* OR enrol* OR retention OR retain*) N4 (survivor* OR patient* OR volunteer* OR participant*))  TI ((Patient* OR criteria OR subject* OR volunteer* OR treatment* OR participant*) N2 selection*) OR AB ((Patient* OR criteria OR subject* OR volunteer* OR treatment* OR participant*) N2 selection*)  TI ((patient* OR volunteer* OR participant*) N2 (compliance OR dropout* OR "drop out*"  OR engag* OR participat* OR refusal)) OR AB ((patient* OR volunteer* OR participant*) N2  (compliance OR dropout* OR "drop out*" OR engag* OR participat* OR refusal))  S4 OR S5 OR S6 OR S7 OR S8  (MH "Exercise+") OR (MH "Physical Activity") OR (MH "Physical Fitness+") OR (MH "Physical  Performance") OR (MH "Sports+") OR (MH "Resistance Training") OR (MH "Therapeutic  Exercise+") OR (MH "Exercise Intensity")  TI (isometric* or isotonic* or isokinetic* OR exercis*) OR AB (isometric* or isotonic* or isokinetic* OR exercis*)  TI ((resistance OR strength* OR weight* OR endurance) N3 (train* OR exercis*)) OR AB  ((resistance OR strength* OR weight* OR endurance) N3 (train* OR exercis*))  TI ((physical* or motion* or cardiopulmonary or cardiorespiratory) N3 (fit* or therap*)) OR  AB ((physical* or motion* or cardiopulmonary or cardiorespiratory) N3 (fit* or therap*)) TI (treadmill* or cross-train* or rowing or sport* OR exercise* OR "physical activit*" OR aerobic* OR run or jog* or running OR walk or walks or walking OR gym* OR pilates OR "recreation* activit*" OR cycling or bicycle or bike or swim*) OR AB (treadmill* or crosstrain* or rowing or sport* OR exercise* OR "physical activit*" OR aerobic* OR run or jog* or running OR walk or walks or walking OR gym* OR pilates OR "recreation* activit*" OR cycling or bicycle or bike or swim*)  TI (circuit* N1 train*) OR AB (circuit* N1 train*)  TI (keep* N1 (active or fit)) OR AB (keep* N1 (active or fit))  S10 OR S11 OR S12 OR S13 OR S14 OR S15 OR S16  TX allocat* random* OR (MH "Quantitative Studies") OR (MH "Placebos") OR TX placebo*  OR TX random* allocat* OR (MH "Random Assignment") OR TX randomi* control* trial* OR TX ( (singl* n1 blind*) OR (singl* n1 mask*) ) OR TX ( (doubl* n1 blind*) OR (doubl* n1 mask*) ) OR TX ( (tripl* n1 blind*) OR (tripl* n1 mask*) ) OR TX ( (trebl* n1 blind*) OR (trebl*  n1 mask*) ) OR TX clinic* n1 trial* OR PT Clinical trial OR (MH "Clinical Trials+")  S3 AND S9 AND S17 AND S18 |
| Web of Science – Core Collection [6461] | (cancer* or neoplas* or tumor* or tumour* or malignan* or carcinoma* or metasta* or oncolog* or leukemi* or leukaemi* or lymphoma* or myeloma* or sarcoma*)    ((recruit* OR enrol* OR retention OR retain* OR adhere* OR compliance) NEAR/4 (strateg* OR plan* OR program* OR barrier* OR facilitator* OR rate OR rate*)) OR ((recruit* OR enrol* OR retention OR retain*) NEAR/4 (survivor* OR patient* OR volunteer* OR participant*)) OR ((Patient* OR criteria OR subject* OR volunteer* OR treatment* OR participant*) NEAR/2 selection*) OR ((patient* OR volunteer* OR participant*) NEAR/4 (compliance OR dropout* OR "drop out*" OR engag* OR participat* OR refusal))    (strength* or isometric* or isotonic* or isokinetic* OR exercis*) OR (resistance NEAR/3 train*) OR ((physical* or motion* or cardiopulmonary or cardiorespiratory) NEAR/3 (fit* or therap*)) OR (treadmill* or cross-train* or rowing or sport* OR exercise* OR "physical activit*" OR aerobic* OR run or jog* or running OR walk or walks or walking OR gym* OR yoga OR pilates OR "recreation* activit*" OR zumba OR salsa* OR cycling or bicycle or bike  OR swim* OR dance OR dancer* OR dances OR dancing) OR (circuit* NEAR/1 train*) OR (keep* NEAR/1 (active or fit))    ("clinical trial*" OR "research design" OR "comparative stud*" OR "evaluation stud*" OR  "controlled trial*" OR "follow-up stud*" OR "prospective stud*" OR random* OR placebo*  OR "single blind*" OR "double blind*")    #1 AND #2 AND #3 AND #4 |
| Cochrane Central Register of Controlled Trials (CENTRAL) [4284] | [mh "Neoplasms"] OR [mh "Cancer Survivors"]  (cancer* or neoplas* or tumor* or tumour* or malignan* or carcinoma* or metasta* or oncolog* or leukemi* or leukaemi* or lymphoma* or myeloma* or sarcoma*):ti,ab,kw #1 OR #2  [mh "Treatment Adherence and Compliance"] OR [mh "Patient Selection"] OR [mh "Patient Compliance"] OR [mh "Patient Dropouts"] OR [mh "Patient Participation"] OR [mh "Patient Satisfaction"]  ((recruit* OR enrol* OR retention OR retain* OR adhere* OR compliance) NEAR/5 (strateg*  OR plan* OR program* OR barrier* OR facilitator* OR rate OR rate*)):ti,ab,kw  ((recruit* OR enrol* OR retention OR retain*) NEAR/5 (survivor* OR patient* OR volunteer* OR participant*)):ti,ab,kw  ((Patient* OR criteria OR subject* OR volunteer* OR treatment* OR participant*) NEAR/2 selection*):ti,ab,kw  ((patient* OR volunteer* OR participant*) NEAR/3 (compliance OR dropout* OR 'drop out'  OR engag* OR participat* OR refusal)):ti,ab,kw  #4 OR #5 OR #6 OR #7 OR #8  [mh "Exercise"] OR [mh "Exercise Therapy"] OR [mh "Physical Fitness"] OR [mh "physical education and training"] OR [mh "Exercise Movement Techniques"] OR [mh "physical endurance"] OR [mh "exercise tolerance"] OR [mh "Physical Exertion"] OR [mh "Sports"] OR [mh "Dancing"]  (strength* or isometric* or isotonic* or isokinetic* OR exercis*):ti,ab,kw  (resistance NEAR/3 train*):ti,ab,kw  ((physical* or motion* or cardiopulmonary or cardiorespiratory) NEAR/3 (fit* or therap*)):ti,ab,kw  (treadmill* or cross-train* or rowing or sport* OR exercise* OR ‘physical activit*’ OR aerobic* OR run or jog* or running OR walk or walks or walking OR gym* OR yoga oR pilates OR ‘recreation* activit*’ OR zumba or salsa* OR cycling or bicycle or bike or swim* or dance or dancer* or dances or dancing):ti,ab,kw (circuit* NEAR/1 train*):ti,ab,kw  (keep* NEAR/1 (active or fit)):ti,ab,kw  #10 OR #11 OR #12 OR #13 OR #14 OR #15 OR #16  #3 AND #9 AND #17 |
