# Supplementary material for: Retention rates and reasons for non-retention in exercise oncology trials in the post-treatment phase—a systematic review
Source: J Cancer Surviv. 2024 Apr 3;19(5):1535–43. doi: 10.1007/s11764-024-01569-4 (PMC12460456; doi:10.1007/s11764-024-01569-4)
Supplement: Supplementary file 2 — Supplementary file2 (DOCX 131 KB) [file 11764_2024_1569_MOESM2_ESM.docx]

**Supplemental Material 2 – Details of Individual Studies by Cancer Type**

*Title:* Retention Rates and Reasons for Non-Retention in Exercise Oncology Trials in the Post-Treatment Phase- A Systematic Review

*Authors*: Sofia Hu^1^, David Mockler^4^, Emer Guinan^2,3^, Linda O’Neill^2,3^

*Affiliations*:

1. School of Pharmacy and Pharmaceutical Sciences, Trinity College Dublin, University of Dublin, Dublin, Ireland
2. Trinity St. James’s Cancer Institute, Dublin, Ireland.
3. Discipline of Physiotherapy, School of Medicine, Trinity College Dublin, University of Dublin, Dublin, Ireland.
4. John Stearne Library, Trinity Centre for Health Sciences, St. James’s Hospital, Dublin, Ireland.

*Corresponding Author:* Dr Linda O’Neill, Research Fellow, Trinity St James’s Cancer Institute, Dublin 8, Ireland. Email: [loneill4@tcd.ie](mailto:loneill4@tcd.ie)

**Breast**

| **Author (Country)** | **Sample size (n)** | **Gender (F:M, %)** | **Cancer Types (%)** | **Mean Age (Years)** | **Exercise Intervention(s)** | **Usual Care Control (Y/N)** | **Duration of Intervention** | **Duration of Trial Participation** | **Total Number of Study Assessments** | **Timing of Study Assessments** | **Retention Rate Immediately Post-Intervention (%)** |
| --- | --- | --- | --- | --- | --- | --- | --- | --- | --- | --- | --- |
| Ahmed, 2006 (US)[1] | 85 | 100:0 | Breast (100) | Intervention: 52.3 (7.7) Control: 51.7 (7.5) | Intervention: RET  Control: UC | Yes | 6 months | 6 months | 2 | Baseline, 6 months (End of Intervention) | *Overall:* 52.94  *Intervention:* 54.76  *Control:* 51.16 |
| Ammitzboll, 2019 (Denmark) [2] | 158 | 100:0 | Breast (100) | Intervention: 53 (Range: 33-73) Control: 52 (30-74) | Intervention: RET Control: UC | Yes | 20 weeks | 50 weeks | 3 | Baseline, 20 weeks (End of Supervised Intervention), 50 weeks (End of Unsupervised Intervention) | *Overall:* 82.28  *Intervention:* 82.93  *Control:* 81.58 |
| Anderson, 2012 (US) [3] | 104 | 100:0 | Breast (100) | Age Groups: Intervention: <50: 40, 50 to <65: 44, 65 to <75: 8, >75: 8; Control: <50: 44, 50 to <65: 37, 65 to <75: 13, >75: 6 | Intervention: AET & RET Control: UC | Yes | 6 months (Initial 3 month Intensive Phase) | 6 months | 7 | Baseline, 3 months (End of Intensive Phase), 6 months (End of Intervention), 9, 12, 15, 18 months (post enrolment) | *Overall:* 76.92  *Intervention:* 82.69  *Control:* 71.15 |
| Artese, 2020 (US) [4] | 44 | 100:0 | Breast (100) | 60.3 (8.3) | Intervention: AET & RET Control: Yin yoga | No | 24 weeks | 24 weeks | 2 | Baseline, 24 weeks (End of Intervention) | *Overall:* 72.73 *Intervention:* 76.19 *Control:* 69.57 |
| Barbosa, 2021 (Brazil) [5] | 60 | 100:0 | Breast (100) | Circuit Training: 54.0 Pilates: 52.0 Control: 59.8 | Interventions: Circuit Training, Pilates Control: UC | Yes | 8 weeks | 8 weeks | 2 | Baseline, 8 weeks (End of Intervention) | *Overall:* 80 *Intervention:* Circuit Training: 75, Pilates: 75 *Control:* 90 |
| Cantarero-Villanueva, 2011 (Spain) [6] | 78 | 100:0 | Breast (100) | Intervention: 49 (9) Control: 48 (9) | Intervention: RET Control: UC | Yes | 8 weeks | 8 months | 3 | Baseline, 8 weeks (End of Intervention), 6 months (from end of intervention) | *Overall:* 85.9 *Intervention:* 84.21 *Control:*  87.5 |
| Casla, 2015 (Spain) [7] | 94 | 100:0 | Breast (100) | Intervention: 45.91 (8.21) Control: 51.87 (8.21) | Intervention: AET & RET Control: UC | Yes | 12 weeks | 9 months | 3 | Baseline, 12 weeks (End of Intervention), 6 months (from end of intervention (intervention group only)) | *Overall:* 94.68 *Intervention:* 93.61 *Control:*  95.74 |
| Daley, 2007 (UK) [8] | 108 | 100:0 | Breast (100) | Intervention: 51.6 (8.8), Placebo: 50.6 (8.7) UC: 51.1 (8.6) | Intervention: AET Controls: Placebo Exercise, UC | Yes | 8 weeks | 24 weeks | 3 | Baseline, 8 weeks (End of Intervention), 24 weeks (from baseline) | *Overall:* 94.44 *Intervention:* 97.06 *Control:*  Placebo: 100, UC: 86.84 |
| Dieli-Conwright, 2018 (US) [9] | 100 | 100:0 | Breast (100) | Intervention: 52.8 (10.6) Control: 53.6 (10.1) | Intervention: AET & RET Control: UC | Yes | 16 weeks | 7 months | 3 | Baseline, 16 weeks (End of Intervention), 3 months (from end of intervention; exercise group only) | *Overall:* 93 *Intervention:* 96  *Control:* 90 |
| Fields, 2016 (UK) [10] | 40 | 100:0 | Breast (100) | Intervention: 60 (8) Control: 66 (7) | Intervention: Nordic Walking  Control: UC | Yes | 12 weeks | 12 weeks | 3 | Baseline, 6 weeks (During Intervention; end of supervised intervention), 12 weeks (End of intervention; end of independent exercise) | *Overall:* 100 *Intervention:* 100  *Control:* 100 |
| Greenlee, 2013 (US) [11] | 42 | 100:0 | Breast (100) | Intervention: 52.6 (8.0) Control: 48.6 (9.6) | Intervention: AET  Control: UC | Yes | 6 months | 12months | 5 | Baseline, 3 months (during intervention), 6 months (end of intervention), 9 months, 12 months (from baseline) | *Overall:* 90.48 *Intervention:* 95.45 *Control:* 85 |
| Guinan, 2013 (Ireland) [12] | 26 | 100:0 | Breast (100) | Intervention: 50.05 (8.27) Control: 45.05 (9.04) | Intervention: AET Control: UC | Yes | 8 weeks | 5 months | 3 | Baseline, 8 weeks (End of Intervention), 3 months (from end of intervention) | *Overall:* 84.62 *Intervention:* 87.5 *Control:* 80 |
| Hagstrom, 2016 (Australia) [13] | 39 | 100:0 | Breast (100) | Intervention: 51.2 (8.5) Control: 52.7 (9.4) | Intervention: RET Control: UC | Yes | 16 weeks | 17 weeks | 2 | Baseline, 17 weeks (from baseline) | *Overall:* 87.18 *Intervention:* 95  *Control:* 78.95 |
| Irwin, 2008 (US) [14] | 75 | 100:0 | Breast (100) | Intervention: 56.5 (9.5) Control: 55.1 (7.7) | Intervention: AET Control: UC | Yes | 6 months | 6 months | 2 | Baseline, 6 months (End of Intervention) [Intervention group 7-day PA logs assessed weekly] | *Overall:* 89.33 *Intervention:* 91.89 *Control:* 86.84 |
| Irwin, 2015 (US) [15] | 96 | 100:0 | Breast (100) | Intervention: 62 (7) Control: 60.5 (7) | Intervention: AET and RET Control: UC | Yes | 12 months | 12 months | 5 | Baseline, 3, 6, 9 months (During Intervention), 12 months (End of Intervention) | *Overall:* 84.375 *Intervention:* 93.75 *Control: 79.17* |
| Jones, 2020 (New Zealand) [16] | 51 | 100:0 | Breast (100) | Intervention: 55.8 (7.2) Control: 55.9 (7.1) | Intervention: AET & RET Control: UC | Yes | 12 weeks | 13 weeks | 2 | Baseline, 13 weeks (from baseline) | *Overall:* 94.4 *Intervention:* 100  *Control:* 86.21 |
| Kaltsatou, 2010 (Greece) [17] | 27 | 100:0 | Breast (100) | Intervention: 56.6 (4.2) Control: 57.1 (4.1) | Intervention: AET (Greek Traditional Dancing) Control: UC | Yes | 24 weeks | 24 weeks | 2 | Baseline, 24 weeks (End of Intervention) | *Overall:* 100 *Intervention:* 100  *Control:* 100 |
| Kilbreath, 2020 (Australia) [18] | 88 | 100:0 | Breast (100) | Intervention: 53.7 (10.4) Control: 59.5 (8.0) | Intervention: AET & RET Control: UC | Yes | 12 weeks | 12 weeks | 2 | Baseline, 12 weeks (End of Intervention) | *Overall:* 96.59 *Intervention:* 95.12 *Control:*  97.87 |
| McNeil, 2019 (Canada) [19] | 45 | 100:0 | Breast (100) | HI: 57.7 (10.4) LI 57.7 (9.3) Control: 60.1 (8.5) | Interventions: HI, LI AET, Control: UC | Yes | 12 weeks | 24 weeks | 3 | Baseline, 12 weeks (End of Intervention), 24 weeks (from baseline) | *Overall:* 95.56 *Intervention:* HI: 100 LI: 100  *Control:* 86.67 |
| Milne, 2008 (Australia) [20] | 58 | 100:0 | Breast (100) | Intervention: 55.2 (8.4) Control: 55.1 (8.0) | Intervention: AET & RET Control: UC | Yes | 12 weeks | 24 weeks | 4 | Baseline, 12 weeks (end of IG intervention), 18 weeks (from baseline), 24 weeks (end of WLC intervention) | *Overall:* 100 *Intervention:* 100  *Control:*  100 |
| Northey, 2019 (Australia) [21] | 17 | 100:0 | Breast (100) | Intervention (HIIT): 60 (8.12) Intervention (CMIT): 65 (7.68) Control: 61 (7.92) | Intervention: AET (HIIT), AET (CMIT) Control: UC | Yes | 12 weeks | 12 weeks | 2 | Baseline, 12 weeks (end of intervention) | *Overall:* 100 *Intervention:* 100  *Control:* 100 |
| Odynets, 2018 (Ukraine) [22] | 124 | 100:0 | Breast (100) | 59.49 (1.06) | Interventions: Aqua/Recreational Aerobics & Conditional Swimming,Conditional Swimming & Pilates, Yoga | No | 48 weeks | 48 weeks | 2 | Baseline, 48 weeks (End of Intervention) | *Overall:* 92.74 *Intervention:*  Aqua Aerobics: 90 Pilates: 90.91 Yoga: 100  *Control:* N/A |
| Owusu, 2022 (US) [23] | 213 | 100:0 | Breast (100) | Intervention: 71.81 (5.99) Control: 71.95 (5.82) | Intervention: AET & RET & Fitbit Control: Support group & Fitbit | No | 20 weeks | 20 weeks | 2 | Baseline, 20 weeks (End of Intervention) | *Overall:* 90.14 *Intervention:*  87.04 *Control:* 93.33 |
| Pinto, 2003 (US) [24, 25] | 24 | 100:0 | Breast (100) | 52.5 (6.8) | Intervention: AET & RET (RET only introduced in last month of the 3 month intervention) Control: UC | Yes | 12 weeks | 12 weeks | 2 | Baseline, 12 weeks (end of intervention; intervention group only) | *Overall/Control:* N/A (only intervention group completed post-intervention assessment)  *Intervention:* 100 |
| Pinto, 2005 (US) | 86 | 100:0 | Breast (100) | Intervention: 53.42 (9.08) Control: 52.86 (10.38) | Intervention: Physical Activity Control: UC | Yes | 12 weeks | 36 weeks | 2 | Baseline, 12 weeks (End of Intervention) | *Overall:* 95.35 *Intervention:* 90.7  *Control:* 100 |
| Portela, 2008 (US) [26] | 34 | 100:0 | Breast (100) | Gym: 49.8 (6.9) Home: 51.2 (7.3) Control: 59.6 (16.7) | Intervention: AET & RET: Gym, Home Control: UC | Yes | 26 weeks | 26 weeks | 3 | Baseline, 13 weeks (During Intervention), 26 weeks (End of Intervention) | *Overall:* 70.59 *Intervention:* Gym: 66.67 Home: 53.85 *Control:* 100 |
| Rogers, 2014 (US) [27] | 46 | 100:0 | Breast (100) | Intervention: 57.2 (5.5) Control: 55.2 (9.1) | Intervention: AET & RET Control: UC | Yes | 3 months | 3 months | 2 | Baseline, 3 months (End of Intervention) | *Overall: 95.65 Intervention:* 95.45 *Control: 95.83* |
| Saarto, 2012 (Finland) [28] | 573 | 100:0 | Breast (100) | Intervention: 52.3 [Range 36-68] Control: 52.4 [Range 35-68] | Intervention: AET (Step Aerobics & Circuit Training) Control: UC | Yes | 12 months | 12 months | 3 | Baseline, 6 months (During Intervention), 12 months (End of Intervention) | *Overall:* 87.26 *Intervention:* 87.09  *Control:* 87.45 |
| Sagen, 2009 (Norway) [29] | 204 | 100:0 | Breast (100) | Intervention: 54 (90.6) Control: 55 (90.5) | Intervention: RET Control: UC (Activity Restriction) | Yes | 6 months | 2 years | 4 | Baseline, 3 months (During Intervention), 6 months (End of Intervention), 2 years (after surgery) | *Overall:* 91.67 *Intervention:* 87.5  *Control:* 97 |
| Santos, 2019 (Brazil) [30] | 26 | 100:0 | Breast (100) | Intervention: 55.0 (5.8) Control: 54.3 (5.2) | Intervention: RET Control: UC | Yes | 8 weeks | 8 weeks | 2 | Baseline, 8 weeks (End of Intervention) | *Overall:* 96.15 *Intervention:* 92.31  *Control:* 100 |
| Schmidt, 2012 (Germany) [31] | 38 | 100:0 | Breast (100) | Intervention: 58 (8.41) Control: 55 (10.59) | Intervention: RET Control: Gymnastics | No | 6 months | 6 months | 3 | Baseline, 3 months (During Intervention), 6 months (End of Intervention) | *Overall:* 86.84 *Intervention:* 73.68  *Control:* 94.74 |
| Schmitz, 2009 (US) [32] | 141 | 100:0 | Breast (100) | Intervention: 56 (9) Control: 58 (10) | Intervention: RET Control: UC | Yes | 12 months | 12 months | 2 | Baseline, 12 months (End of Intervention) | *Overall:* 98.58 *Intervention:* 98.59  *Control:* 98.57 |
| Uhm, 2016 (Korea) [33] | 356 | 100:0 | Breast (100) | Intervention: 49.3 (8.0) Control: 51.3 (10.7) | Intervention: AET & RET Control: UC | Yes | 12 weeks | 12 weeks | 3 | Baseline, 6 weeks (During Intervention), 12 weeks (End of Intervention) | *Overall:* 95.22 *Intervention:* 93.3  *Control:* 97.18 |
| Winters-Stone, 2011 (US) [34] | 106 | 100:0 | Breast (100) | Intervention: 62.3 (6.7) Control: 62.2 (6.7) | Intervention: RET Control: Flexibility Training | No | 12 months | 12 months | 3 | Baseline, 6 months (During Intervention), 12 months (End of Intervention) | *Overall:* 100 *Intervention: 100*  *Control:* 100 |

**Colon**

| **Author (Country)** | **Sample size (n)** | **Gender (F:M, %)** | **Cancer Types (%)** | **Mean Age (Years)** | **Exercise Intervention(s)** | **Usual Care Control (Y/N)** | **Duration of Intervention** | **Duration of Trial Participation** | **Total Number of Study Assessments** | **Timing of Study Assessments** | **Retention Rate Immediately Post-Intervention (%)** |
| --- | --- | --- | --- | --- | --- | --- | --- | --- | --- | --- | --- |
| Brown, 2018 (US) [35] | 39 | 70:30 | Colon (100) | Intervention (High Dose): 52.1 (11.2) Intervention (Low Dose): 58.4 (9.6) Control: 55.6 (4.3) | Intervention: AET (High Dose), AET (Low Dose) Control: UC | Yes | 6 months | 6 months | 2 | Baseline, 6 months (End of Intervention) | *Overall:* 97.44  *Intervention:*  100  *Control:*  92.31 |

**Colorectal**

| **Author (Country)** | **Sample size (n)** | **Gender (F:M, %)** | **Cancer Types (%)** | **Mean Age (Years)** | **Exercise Intervention(s)** | **Usual Care Control (Y/N)** | **Duration of Intervention** | **Duration of Trial Participation** | **Total Number of Study Assessments** | **Timing of Study Assessments** | **Retention Rate Immediately Post-Intervention (%)** |
| --- | --- | --- | --- | --- | --- | --- | --- | --- | --- | --- | --- |
| Pinto, 2013 (US) [36] | 46 | Intervention: (60:40) Control: (54:46) | Intervention: Colon (55) Rectal: (45) Control: Colon (58) Rectal (42) | Intervention: 59.5 (11.2) Control: 55.6 (8.24) | Intervention: AET Control: UC | Yes | 12 weeks | 12 months | 4 | Baseline, 12 weeks (end of intervention), 6 months (from baseline), 12 months (from baseline ) | *Overall:* 93.48  *Intervention:* 95  *Control:*  92.31 |
| Devin, 2015 (Australia)[37] | 47 | High Intensity AET: (40:60), Moderate Intensity AET: (59.9:40.1) | Colon (74.5), Rectal (25.5) | High Intensity AET: 61.4 (11.1), Moderate Intensity AET: 61.5 (10.8) | Interventions: High Intensity AET, Moderate Intensity AET | No | 4 weeks | 4 weeks | 2 | Baseline, 4 weeks (End of Intervention) | *Overall:* 95.74  *Intervention:*  96.67  *Control:* 94.12 |

**Endometrial**

| **Author (Country)** | **Sample size (n)** | **Gender (F:M, %)** | **Cancer Types (%)** | **Mean Age (Years)** | **Exercise Intervention(s)** | **Usual Care Control (Y/N)** | **Duration of Intervention** | **Duration of Trial Participation** | **Total Number of Study Assessments** | **Timing of Study Assessments** | **Retention Rate Immediately Post-Intervention (%)** |
| --- | --- | --- | --- | --- | --- | --- | --- | --- | --- | --- | --- |
| Gorzelitz, 2021 (US) [38] | 40 | 100:0 | Endometrial (100) | Intervention: 60.9 (9.6) Control: 60.9 (8.0) | Intervention: RET Control: UC | Yes | 10 weeks | 15 weeks | 3 | Baseline, 10 weeks (End of Intervention), 5 weeks (from end of supervised intervention) | *Overall:* 95  *Intervention:* 95  *Control:* 95 |

**Glioma**

| **Author (Country)** | **Sample size (n)** | **Gender (F:M, %)** | **Cancer Types (%)** | **Mean Age (Years)** | **Exercise Intervention(s)** | **Usual Care Control (Y/N)** | **Duration of Intervention** | **Duration of Trial Participation** | **Total Number of Study Assessments** | **Timing of Study Assessments** | **Retention Rate Immediately Post-Intervention (%)** |
| --- | --- | --- | --- | --- | --- | --- | --- | --- | --- | --- | --- |
| Gehring, 2018 (Netherlands) [39] | 34 | Intervention (56:44) Control (55:45) | Glioma (100) | Intervention: 48 (9.4) Control: 48 (11.9) | Intervention: AET Control: UC | Yes | 6 months | 6 months | 2 | Baseline, 6 months (End of Intervention) | *Overall:* 94.12  *Intervention:*  91.3  *Control:* 100 |

**Haematological**

| **Author (Country)** | **Sample size (n)** | **Gender (F:M, %)** | **Cancer Types (%)** | **Mean Age (Years)** | **Exercise Intervention(s)** | **Usual Care Control (Y/N)** | **Duration of Intervention** | **Duration of Trial Participation** | **Total Number of Study Assessments** | **Timing of Study Assessments** | **Retention Rate Immediately Post-Intervention (%)** |
| --- | --- | --- | --- | --- | --- | --- | --- | --- | --- | --- | --- |
| Furzer, 2016 (Australia) [40] | 44 | Not reported | Non-Hodgkins Lymphoma (73) Hodgkins Lymphoma (16) Multiple Myeloma (11) | Intervention: 48.2 (12.3) Control: 49.6 (14.1) | Intervention: AET & RET Control: UC | Yes | 12 weeks | 24 weeks | 3 | Baseline, 12 weeks (End of Intervention), 24 weeks (from baseline) | *Overall:*84.09  *Intervention:* 81.82  *Control:*86.36 |
| Shelton, 2008 (US) [41] | 61 | Supervised: 35:65 Unsupervised: 41:59 | Intervention: AML (31) ALL (7) CML (11) CLL (11) NHL (19) HD (19) Control: AML (44) ALL (7) CML (11) CLL (7) NHL (19) | Supervised: 43.65 (13.18) Unsupervised: 48.93 (11.66) | Interventions: Supervised AET & RET, Unsupervised RET & Walking | No | 4 weeks | 4 weeks | 2 | Baseline, 4 weeks (End of Intervention) | *Overall:* 86.89  *Intervention:* 86.67  *Control:*87.1 |

**Head and Neck**

| **Author (Country)** | **Sample size (n)** | **Gender (F:M, %)** | **Cancer Types (%)** | **Mean Age (Years)** | **Exercise Intervention(s)** | **Usual Care Control (Y/N)** | **Duration of Intervention** | **Duration of Trial Participation** | **Total Number of Study Assessments** | **Timing of Study Assessments** | **Retention Rate Immediately Post-Intervention (%)** |
| --- | --- | --- | --- | --- | --- | --- | --- | --- | --- | --- | --- |
| McNeely, 2015 (Canada) [42] | 52 | 33:67 | Head and Neck (100) | 52 | Intervention: RET (progressive) Control: RET (standard) | Yes | 12 weeks | 12 months | 3 | Baseline, 12 weeks (End of Intervention), 12 months (from baseline) | *Overall:*  92.31  *Intervention:* 92  *Control:* 92.59 |

**Lung**

| **Author (Country)** | **Sample size (n)** | **Gender (F:M, %)** | **Cancer Types (%)** | **Mean Age (Years)** | **Exercise Intervention(s)** | **Usual Care Control (Y/N)** | **Duration of Intervention** | **Duration of Trial Participation** | **Total Number of Study Assessments** | **Timing of Study Assessments** | **Retention Rate Immediately Post-Intervention (%)** |
| --- | --- | --- | --- | --- | --- | --- | --- | --- | --- | --- | --- |
| Brocki, 2014 (Denmark) [43] | 78 | Intervention: (46:54) Control: (35:65) | Lung (100) | Intervention: 64 (10) Control: 65 (9) | Intervention: AET and RET Control: 1 individual instruction in exercise trraining | No | 10 weeks | 12 months | 3 | Baseline, 4 months, 12 months (from baseline) | *Overall:* 75.64  *Intervention:* 68.29  *Control:* 83.78 |

**Mixed Population**

| **Author (Country)** | **Sample size (n)** | **Gender (F:M, %)** | **Cancer Types (%)** | **Mean Age (Years)** | **Exercise Intervention(s)** | **Usual Care Control (Y/N)** | **Duration of Intervention** | **Duration of Trial Participation** | **Total Number of Study Assessments** | **Timing of Study Assessments** | **Retention Rate Immediately Post-Intervention (%)** |
| --- | --- | --- | --- | --- | --- | --- | --- | --- | --- | --- | --- |
| Broderick, 2013 (Ireland) [44] | 43 | Intervention: (96:4) Control: (75:25) | Breast (72.1) Colon (11.6) Lymphoma (7.0) Oesophageal (4.7) Gynaecological (4.7) | Intervention: 52.3 (8.3) Control: 51.2 (10.3) | Intervention: AET Control: UC | Yes | 8 weeks | 5 months | 3 | Baseline, 8 weeks (End of Intervention), 3 months (from end of intervention) | *Overall:* 95.83  *Intervention:* 91.89  *Control:* 100 |
| Gothe, 2020 (US) [45] | 78 | Intervention (AET): 88.5:11.5 Intervention (Yoga): 84.6:15.4 Control: 84.6:15.4 | AET: Breast (55.55), Colorectal (3.70) Liver/bile duct (3.70) Lung (7.41) Lymphoma (7.41) Ovarian (11.11) Prostate (3.70) Skin (3.70) Thyroid (3.70) Yoga: Breast (69.20) cervical (3.80) Endometrial/Uterine (3.80) Multiple myeloma (3.80) Prostate (7.70) Skin (7.70) Thyroid (3.80) Control: Breast (53.80) Cervical (3.80) Leukemia (7.70) Lymphoma (3.80) Sarcoma (3.80) Skin (3.80) | Intervention (AET): 55.92 (9.2) Intervention (Yoga): 55.0 (9.57) Control: 55.88 (10.7) | Intervention: Aerobic Walking, Yoga Control: Stretching-toning | No | 12 weeks | 12 weeks | 2 | Baseline, 12 weeks (End of Intervention) | *Overall:* 74.36  *Intervention:* AET: 65.38 Yoga: 65.38  *Control:* 92.31 |
| Kampshoff, 2015 (Netherlands) [46] | 277 | HI 80:20 LMI 82:18 Control: 78:22 | HI: Breast (68) Colon (17) Ovarian (4) Lymphoma (10) Testis (1) LMI: Breast (65) Colon (20) Ovarian (3) Lymphoma (9) Cervix (2) Control: Breast (63) Colon (17) Ovarian (6) Lymphoma (9) Cervix (2) Testis (4) | HI: 54 (11.0) LMI: 53 (11.3) Control: 54 (10.9) | Interventions: HI, LMI AET & RET Control: UC | Yes | 12 weeks | 12 weeks | 2 | Baseline, 12 weeks (End of Intervention) | *Overall:* 100  *Intervention:* 100  *Control:* 100 |
| Kneis, 2019 (Germany) [47] | 50 | 73:27 | Breast (34) Colorectal (34) Gynecological (10) Upper GI (5) Non-small cell Lung (2) Non-Hodgkin's Lymphoma (12) Multiple myeloma (2) | 62 [MEDIAN] | Intervention: AET & Balance training Control: AET only | No | 12 weeks | 12 weeks | 2 | Baseline, 12 weeks (End of Intervention) | *Overall:* 82  *Intervention:* 80  *Control:* 84 |
| Lapen, 2018 (US) [48] | 42 | 100:0 | Intervention: Breast (87) Ovarian (13) Control: Breast (90) Ovarian (10) | [MEDIAN] Intervention: 58 (IQR 54-62) Control: 55 (IQR 53-60) | Intervention: Vigorous Yoga Control: Restorative Yoga | No | 24 weeks | 24 weeks | 3 | Baseline, 12 weeks (End of supervised intervention), 24 weeks (End of unsupervised intervention) | *Overall:* (End of Supervised Intervention): 78.57  *Intervention:* 61.9  *Control:* 95.24 |
| LaStayo, 2011 (US) [49] | 49 | Intervention: 65:35 Control: 60:40 | Intervention: Breast (50) Prostate (20) Colorectal (20) Lymphoma (5) Lung (5) Control: Breast (60) Prostate (35) Colorectal (15) Lung (10) | Intervention: 75 (7) Control: 73 (5) | Intervention: AET, RET, Stretching & Balance Control: UC | Yes | 12 weeks | 12 weeks | 2 | Baseline, 12 weeks (End of Intervention) | *Overall:* 81.63  *Intervention:* 71.43  *Control:* 95.24 |
| Lin, 2019 (US) [50] | 410 | Intervention: 95:5 Control: 97:3 | Intervention: Breast (75) Hematological (7) Alimentary (4) Gynecological (6) Other (8) Control: Breast (79) Hematological (7) Alimentary (8) Gynecological (3) Other (4) | Intervention: 55 (11.1) Control: 53.7 (9.3) | Intervention: Yoga Control: UC | Yes | 4 weeks | 4 weeks | 2 | Baseline, 4 weeks (End of Intervention) | *Overall:* 78.29  *Intervention:* 89.55  *Control:* 75 |
| Martin, 2015 (Australia) [51] | 159 | 45:55 | Breast (45) Prostate (55) | Prostate: LI 65 (6.3) HI 65.3 (7) Control: 66.9 (6.1) Breast: LI 58.2 (9.6) HI 53.5 (9) Control: 57.2 (9.8) | Interventions: High Intensity, Low intensity AET & RET Control: UC | Yes | 10 weeks | 26 weeks | 3 | Baseline, 10 weeks (End of Intervention), 4 weeks (from end of intervention; intervention group only) | *Overall:* 80.5  *Intervention:* HI: 95 LI: 95.45  *Control:* 64 |
| Rabin, 2015 (US) [52] | 35 | Intervention: 78.9:21.1 Control: 87.5:12.5 | Mixed cohort, diagnoses not reported | Intervention: 33.3 (4.6) Control: 33.9 (3.5) | Intervention: AET Control: UC | Yes | 12 weeks | 24 weeks | 3 | Baseline, 12 weeks (End of intervention for intervention group), 24 weeks (End of intervention for WLC) | *Overall:* 77.14  *Intervention:* 78.95  *Control:* 75 |
| Siebert, 2022 (Germany) [53] | 36 | 60.6:39.4 | Breast (39.4) Colorectal (9.1) Prostate (9.1) Oesophageal (3) Lymphoma (9.1) Carcinoma of unknown primary origin (6.1) Bronchial (3) Ovarian (3) Other (18.1) | 57.6 (9.75) | Interventions: MID AET, VIT AET, MIT RET, VIT RET Control: UC | Yes | 4 weeks | 4 weeks | 2 | Baseline, 4 weeks (End of Intervention) | *Overall:* 91.67  *Intervention:* 92.59  *Control:* 88.89 |
| Thorsen, 2005 (Norway) [54] | 139 | Intervention: 68:32 Control: 65:35 | Intervention: Breast (36) Gynecological (22) Lymphoma (24) Testicular (19) Control: Breast (40) Gynecological (21) Lymphoma (21) Testicular (17) | Intervention: 39 (8.4) Control: 39.1 (8.6) | Intervention: AET or RET (Choice) Control: UC | Yes | 14 weeks | 14 weeks | 2 | Baseline, 14 weeks (End of Intervention) | *Overall:* 79.86  *Intervention:* 85.81  *Control:* 74.29 |
| Van Weert, 2005 (Netherlands) [55] | 81 | 84:16 | Breast (59.3) NHL/M Hodgkins (8.6) Gynecological (7.3) Head&Neck (6.2) Other (18.6) | 51.6 (9.3) | Interventions: Individual Exercise (AET&RET), Sports, Psychoeducation, Information [The 2 groups were all 4 or choice] | No | 15 weeks | 3 months | 3 | Baseline, 15 weeks (End of Intervention), 3 months (from baseline) | *Overall:* 77.78  *Intervention:* N/R  *Control:* N/R |

**Oesophageal**

| **Author (Country)** | **Sample size (n)** | **Gender (F:M, %)** | **Cancer Types (%)** | **Mean Age (Years)** | **Exercise Intervention(s)** | **Usual Care Control (Y/N)** | **Duration of Intervention** | **Duration of Trial Participation** | **Total Number of Study Assessments** | **Timing of Study Assessments** | **Retention Rate Immediately Post-Intervention (%)** |
| --- | --- | --- | --- | --- | --- | --- | --- | --- | --- | --- | --- |
| Van Vulpen, 2021 (Netherlands) [56] | 120 | Intervention: 15:85 Control: 12:88 | Oesophageal (100) | Intervention: 64.3 (7.8) Control: 63.1 (8.5) | Intervention: AET & RET Control: UC | Yes | 12 weeks | 24 weeks | 3 | Baseline, 12 weeks (End of Intervention), 24 weeks (from baseline) | *Overall:* 91.67  *Intervention:* 88.52  *Control:*  94.92 |

**Oesophagogastric**

| **Author (Country)** | **Sample size (n)** | **Gender (F:M, %)** | **Cancer Types (%)** | **Mean Age (Years)** | **Exercise Intervention(s)** | **Usual Care Control (Y/N)** | **Duration of Intervention** | **Duration of Trial Participation** | **Total Number of Study Assessments** | **Timing of Study Assessments** | **Retention Rate Immediately Post-Intervention (%)** |
| --- | --- | --- | --- | --- | --- | --- | --- | --- | --- | --- | --- |
| O’Neill, 2018 (Ireland) [57] | 37 | Intervention: 15:85 Control: 24:76 | Intervention: Esophagus (40) Esophagogastric junction: (55) Gastric: (5) Control: Esophagus (17.6) Esophagogastric junction (76.5) Gastric (5.9) | Intervention: 67 (7.7) Control: 63 (10.1) | Intervention: AET & RET Control: UC | Yes | 12 weeks | 24 weeks | 3 | Baseline, 12 weeks (End of Intervention), 24 weeks (from baseline) | *Overall:* 93.02  *Intervention:* 100  *Control:* 86.36 |

**Ovarian**

| **Author (Country)** | **Sample size (n)** | **Gender (F:M, %)** | **Cancer Types (%)** | **Mean Age (Years)** | **Exercise Intervention(s)** | **Usual Care Control (Y/N)** | **Duration of Intervention** | **Duration of Trial Participation** | **Total Number of Study Assessments** | **Timing of Study Assessments** | **Retention Rate Immediately Post-Intervention (%)** |
| --- | --- | --- | --- | --- | --- | --- | --- | --- | --- | --- | --- |
| Zhou, 2017 (US) [58] | 144 | 100:0 | Ovarian (100) | Intervention: 57.3 (8.8) Control: 57.4 (8.5) | Intervention: AET Control: UC | Yes | 6 months | 6 months | 2 | Baseline, 6 months (End of Intervention) | *Overall:* 78.47  *Intervention:* 82.43  *Control:* 74.29 |

**Prostate**

| **Author (Country)** | **Sample size (n)** | **Gender (F:M, %)** | **Cancer Types (%)** | **Mean Age (Years)** | **Exercise Intervention(s)** | **Usual Care Control (Y/N)** | **Duration of Intervention** | **Duration of Trial Participation** | **Total Number of Study Assessments** | **Timing of Study Assessments** | **Retention Rate Immediately Post-Intervention (%)** |
| --- | --- | --- | --- | --- | --- | --- | --- | --- | --- | --- | --- |
| Alibhai, 2019 (Canada) [59] | 59 | 0:100 | Prostate (100) | PT: 69.2 (7.3), Group: 71.5 (7.2), Home: 69.6 (8.1) | Interventions: AET & RET delivered by PT, in Groups, at Home | No | 6 months | 6 months | 5 | Baseline, 3 months (During Intervention), 6 months (End of Intervention) | *Overall:*71.19  *Intervention:* PT: 85, Group: 57.89, Home: 70  *Control:* N/A |
| Bourke, 2011 (UK) [60] | 50 | 0:100 | Prostate (100) | Intervention: 71.3 (6.4) Control: 72.2 (7.7) | Intervention: AET and RET Control: UC | *Yes* | 12 weeks | 6 months | 3 | Baseline, 12 weeks (End of Intervention), 6 months (post-enrolment) | *Overall:* 86  *Intervention:* 84  *Control:* 88 |
| Campo, 2014 (US) [61] | 40 | 0:100 | Prostate (100) | [MEDIAN] Intervention: 72 (58-90) Control: 73 [61-93] | Intervention: Qigong  Control: Stretching | No | 12 weeks | 13 weeks | 2 | Baseline, 13 weeks (1 week post-end of intervention) | *Overall:* 72.5  *Intervention:* 80  *Control:* 65 |
| Cormie, 2013 (Australia) [62] | 57 | 0:100 | Prostate (100) | Intervention: 69.5 (7.3) Control: 70.1 (7.3) | Intervention: AET and RET Control: UC | Yes | 12 weeks | 12 weeks | 2 | Baseline, 12 weeks (End of Intervention) | *Overall:* 96.49  *Intervention:* 96.55  *Control:* 96.43 |
| Kim, 2018 (Korea) [63] | 51 | 0:100 | Prostate (100) | Intervention: 70.5 (5) Control: 71 (5.5) | Intervention: AET & RET Control: Stretching (placebo) | No | 6 months | 6 months | 2 | Baseline, 6 months (End of Intervention) | *Overall:* 80.39  *Intervention:* 88.46  *Control:* 62 |
| Livingston, 2015 (Australia) [64] | 147 | 0:100 | Prostate (100) | Intervention: 66.9 (7.2) Control: 64.7 (8.7) | Intervention: AET & RET Control: UC | Yes | 12 weeks | 12 weeks | 2 | Baseline, 12 weeks (End of Intervention) | *Overall:* 88.44  *Intervention:* 87.04  *Control:* 89.25 |
| Nilsen, 2015 (Norway) [65] | 58 | 0:100 | Prostate (100) | Intervention: 66 (6.6) Control: 66 (5) | Intervention: RET Control: UC | Yes | 16 weeks | 16 weeks | 2 | Baseline, 16 weeks (End of Intervention) | *Overall:* 100  *Intervention:* 100  *Control:* 100 |
| Papadopoulos, 2021 (Canada) [66] | 18 | 0:100 | Prostate (100) | RT: 63.2 (6.0) HIIT: 62.0 (10.4) UC: 62.1 (3.2) | Interventions: AET (HIIT), RET Control: UC | Yes | 8 weeks | 8 weeks | 2 | Baseline, 8 weeks (End of Intervention) | *Overall:* 88.89  *Intervention:* RT: 85.71 HIIT: 100  *Control:* 88.33 |
| SantaMina, 2013 (Canada) [67] | 66 | 0:100 | Prostate (100) | Intervention: 72.1 (8.9) Control: 70.6 (9.5) | Interventions: AET, RET | No | 6 months | 12 months | 4 | Baseline, 3 months (During Intervention), 6 months (End of Intervention), 12 months (from baseline) | *Overall:* 66.67  *Intervention:* 75  *Control:* 58.82 |

AET= Aerobic Exercise Training; RET= Resistance Exercise Training; PA= Physical Activity; CBT= Cognitive Behavioural Therapy; HI= High Intensity; LI= Low Intensity; LMI= Low-Moderate Intensity; HIIT= High Intensity Interval Training; CMIT= Continuous Moderate Intensity Training; VIT= Vigorous Intensity Training; MIT= Moderate Intensity Training; WLC= Wait-List Control; AML= Acute Myocytic Leukemia; ALL= Acute Lymphocytic Leukemia; CML= Chronic Myocytic Leukemia; CLL= Chronic Lymphocytic Leukemia; NHL= Non-Hodgkin’s Lymphoma; HD= Hodgkin’s Disease; N/R= Not reported

**References:**

1. Ahmed RL, Thomas W, Yee D, Schmitz KH: **Randomized controlled trial of weight training and lymphedema in breast cancer survivors**. *Journal of Clinical Oncology* 2006, **24**(18):2765-2772.

2. Ammitzbøll G, Johansen C, Lanng C, Andersen EW, Kroman N, Zerahn B, Hyldegaard O, Wittenkamp MC, Dalton SO: **Progressive resistance training to prevent arm lymphedema in the first year after breast cancer surgery: results of a randomized controlled trial**. *Cancer* 2019, **125**(10):1683‐1692.

3. Anderson RT, Kimmick GG, McCoy TP, Hopkins J, Levine E, Miller G, Ribisl P, Mihalko SL: **A randomized trial of exercise on well-being and function following breast cancer surgery: The RESTORE trial**. *Journal of Cancer Survivorship* 2012, **6**(2):172-181.

4. Artese AL, Hunt RL, Ormsbee MJ, Kim JS, Arjmandi BH, Panton LB: **Effect of Functional Impact Training on Body Composition, Bone Mineral Density, and Strength in Breast Cancer Survivors**. *Medicine and Science in Sports and Exercise* 2021, **53**(1):90‐101.

5. Barbosa KP, da Silva LGT, Garcia PA, Freitas CA, da Silva ECF, Pereira TV, Alves AT, Matheus LBG: **Effectiveness of Pilates and circuit-based exercise in reducing arthralgia in women during hormone therapy for breast cancer: a randomized, controlled trial**. *Supportive Care in Cancer* 2021, **29**(10):6051-6059.

6. Cantarero-Villanueva I, Fernández-Lao C, Díaz-Rodriguez L, Fernández-de-las-Peñas C, Del Moral-Avila R, Arroyo-Morales M: **A multimodal exercise program and multimedia support reduce cancer-related fatigue in breast cancer survivors: A randomised controlled clinical trial**. *European Journal of Integrative Medicine* 2011, **3**(3):e189-e200.

7. Casla S, Lopez-Tarruella S, Jerez Y, Marquez-Rodas I, Galvao DA, Newton RU, Cubedo R, Calvo I, Sampedro J, Barakat R *et al*: **Supervised physical exercise improves VO2max, quality of life, and health in early stage breast cancer patients: a randomized controlled trial**. *Breast Cancer Research & Treatment* 2015, **153**(2):371-382.

8. Daley AJ, Crank H, Saxton JM, Mutrie N, Coleman R, Roalfe A: **Randomized trial of exercise therapy in women treated for breast cancer**. *Journal of Clinical Oncology* 2007, **25**(13):1713-1721.

9. Dieli-Conwright CM, Courneya KS, Demark-Wahnefried W, Sami N, Lee K, Buchanan TA, Spicer DV, Tripathy D, Bernstein L, Mortimer JE: **Effects of aerobic and resistance exercise on metabolic syndrome, sarcopenic obesity, and circulating biomarkers in overweight or obese survivors of breast cancer: A randomized controlled trial**. *Journal of Clinical Oncology* 2018, **36**(9):875-883.

10. Fields J, Richardson A, Hopkinson J, Fenlon D: **Nordic Walking as an Exercise Intervention to Reduce Pain in Women With Aromatase Inhibitor–Associated Arthralgia: A Feasibility Study**. *Journal of Pain and Symptom Management* 2016, **52**(4):548-559.

11. Greenlee HA, Crew KD, Mata JM, McKinley PS, Rundle AG, Zhang W, Liao Y, Tsai WY, Hershman DL: **A pilot randomized controlled trial of a commercial diet and exercise weight loss program in minority breast cancer survivors**. *Obesity* 2013, **21**(1):65-76.

12. Guinan E, Hussey J, Broderick JM, Lithander FE, O'Donnell D, Kennedy MJ, Connolly EM: **The effect of aerobic exercise on metabolic and inflammatory markers in breast cancer survivors--a pilot study**. *Supportive care in cancer : official journal of the Multinational Association of Supportive Care in Cancer* 2013, **21**(7):1983-1992.

13. Hagstrom AD, Marshall PWM, Lonsdale C, Papalia S, Cheema BS, Toben C, Baune BT, Fiatarone Singh MA, Green S: **The effect of resistance training on markers of immune function and inflammation in previously sedentary women recovering from breast cancer: a randomized controlled trial**. *Breast Cancer Research and Treatment* 2016, **155**(3):471-482.

14. Irwin ML, Cadmus L, Alvarez-Reeves M, O'Neil M, Mierzejewski E, Latka R, Yu H, DiPietro L, Jones B, Knobf MT *et al*: **Recruiting and retaining breast cancer survivors into a randomized controlled exercise trial: The Yale Exercise and Survivorship Study**. *Cancer* 2008, **112**(11 SUPPL.):2593-2606.

15. Irwin ML, Cartmel B, Gross CP, Ercolano E, Li F, Yao X, Fiellin M, Capozza S, Rothbard M, Zhou Y *et al*: **Randomized exercise trial of aromatase inhibitor-induced arthralgia in breast cancer survivors**. *Journal of clinical oncology : official journal of the American Society of Clinical Oncology* 2015, **33**(10):1104-1111.

16. Jones LM, Stoner L, Baldi JC, McLaren B: **Circuit resistance training and cardiovascular health in breast cancer survivors**. *European Journal of Cancer Care* 2020, **29**(4):e13231.

17. Kaltsatou A, Mameletzi D, Douka S: **Physical and psychological benefits of a 24-week traditional dance program in breast cancer survivors**. *Journal of Bodywork & Movement Therapies* 2011, **15**(2):162-167.

18. Kilbreath SL, Ward LC, Davis GM, Degnim AC, Hackett DA, Skinner TL, Black D: **Reduction of breast lymphoedema secondary to breast cancer: a randomised controlled exercise trial**. *Breast Cancer Research and Treatment* 2020, **184**(2):459-467.

19. McNeil J, Brenner DR, Stone CR, O'Reilly R, Ruan Y, Vallance JK, Courneya KS, Thorpe KE, Klein DJ, Friedenreich CM: **Activity Tracker to Prescribe Various Exercise Intensities in Breast Cancer Survivors**. *Med Sci Sports Exerc* 2019, **51**(5):930-940.

20. Milne HM, Wallman KE, Gordon S, Courneya KS: **Effects of a combined aerobic and resistance exercise program in breast cancer survivors: a randomized controlled trial**. *Breast Cancer Research & Treatment* 2008, **108**(2):279-288.

21. Northey JM, Pumpa KL, Quinlan C, Ikin A, Toohey K, Smee DJ, Rattray B: **Cognition in breast cancer survivors: A pilot study of interval and continuous exercise**. *Journal of Science and Medicine in Sport* 2019, **22**(5):580-585.

22. Odynets T, Briskin Y, Putrov S: **Effectiveness of individualised intervention on pulmonary function in women with post-mastectomy syndrome**. *Physiotherapy Practice & Research* 2018, **39**(2):147-154.

23. Owusu C, Nock NL, Feuntes V, Margevicius S, Hergenroeder P, Austin K, Bennet E, Cerne S, Moore HCF, Petkac J *et al*: **IMPROVE, a community-based exercise intervention versus support group to improve functional and health outcomes among older African American and Non-Hispanic White breast cancer survivors from diverse socioeconomic backgrounds: Recruitment strategies and baseline characteristics**. *Cancer* 2021, **127**(11):1836-1846.

24. Pinto BM, Clark MM, Maruyama NC, Feder SI: **Psychological and fitness changes associated with exercise participation among women with breast cancer**. *Psycho-oncology* 2003, **12**(2):118-126.

25. Pinto BM, Frierson GM, Rabin C, Trunzo JJ, Marcus BH: **Home-based physical activity intervention for breast cancer patients**. *Journal of clinical oncology : official journal of the American Society of Clinical Oncology* 2005, **23**(15):3577-3587.

26. Portela ALM, Santaella CLC, Gómez CC, Burch A: **Feasibility of an exercise program for Puerto Rican women who are breast cancer survivors**. *Rehabilitation Oncology* 2008, **26**(2):20-31.

27. Rogers LQ, Vicari S, Trammell R, Hopkins-Price P, Fogleman A, Spenner A, Rao K, Courneya KS, Hoelzer KS, Robbs R *et al*: **Biobehavioral factors mediate exercise effects on fatigue in breast cancer survivors**. *Medicine and Science in Sports and Exercise* 2014, **46**(6):1077-1088.

28. Saarto T, Sievänen H, Kellokumpu-Lehtinen P, Nikander R, Vehmanen L, Huovinen R, Kautiainen H, Järvenpää S, Penttinen HM, Utriainen M *et al*: **Effect of supervised and home exercise training on bone mineral density among breast cancer patients. A 12-month randomised controlled trial**. *Osteoporosis international : a journal established as result of cooperation between the European Foundation for Osteoporosis and the National Osteoporosis Foundation of the USA* 2012, **23**(5):1601-1612.

29. Sagen Å, Kåresen R, Risberg MA: **Physical activity for the affected limb and arm lymphedema after breast cancer surgery. A prospective, randomized controlled trial with two years follow-up**. *Acta Oncologica* 2009, **48**(8):1102-1110.

30. Santos WDND, Vieira A, de Lira CAB, Mota JF, Gentil P, de Freitas Junior R, Battaglini CL, Bottaro M, Vieira CA: **Once a Week Resistance Training Improves Muscular Strength in Breast Cancer Survivors: A Randomized Controlled Trial**. *Integrative cancer therapies* 2019, **18**:1534735419879748.

31. Schmidt T, Weisser B, Jonat W, Baumann FT, Mundhenke C: **Gentle strength training in rehabilitation of breast cancer patients compared to conventional therapy**. *Anticancer Research* 2012, **32**(8):3229‐3233.

32. Schmitz KH, Troxel AB, Cheville A, Grant LL, Bryan CJ, Gross CR, Lytle LA, Ahmed RL: **Physical Activity and Lymphedema (the PAL trial): assessing the safety of progressive strength training in breast cancer survivors**. *Contemp Clin Trials* 2009, **30**(3):233-245.

33. Uhm KE, Yoo JS, Chung SH, Lee JD, Lee I, Kim JI, Lee SK, Nam SJ, Park YH, Lee JY *et al*: **Effects of exercise intervention in breast cancer patients: is mobile health (mHealth) with pedometer more effective than conventional program using brochure?** *Breast Cancer Research and Treatment* 2017, **161**(3):443-452.

34. Winters-Stone KM, Dobek J, Nail L, Bennett JA, Leo MC, Naik A, Schwartz A: **Strength training stops bone loss and builds muscle in postmenopausal breast cancer survivors: a randomized, controlled trial**. *Breast Cancer Res Treat* 2011, **127**(2):447-456.

35. Brown JC, Damjanov N, Courneya KS, Troxel AB, Zemel BS, Rickels MR, Ky B, Rhim AD, Rustgi AK, Schmitz KH: **A randomized dose-response trial of aerobic exercise and health-related quality of life in colon cancer survivors**. *Psycho-oncology* 2018, **27**(4):1221-1228.

36. Pinto BM, Papandonatos GD, Goldstein MG, Marcus BH, Farrell N: **Home-based physical activity intervention for colorectal cancer survivors**. *Psycho-oncology* 2013, **22**(1):54-64.

37. Devin JL, Sax AT, Hughes GI, Jenkins DG, Aitken JF, Chambers SK, Dunn JC, Bolam KA, Skinner TL: **The influence of high-intensity compared with moderate-intensity exercise training on cardiorespiratory fitness and body composition in colorectal cancer survivors: a randomised controlled trial**. *J Cancer Surviv* 2016, **10**(3):467-479.

38. Gorzelitz J, Costanzo E, Gangnon R, Koltyn K, Dietz AT, Spencer RJ, Rash J, Cadmus-Bertram L: **Feasibility and acceptability of home-based strength training in endometrial cancer survivors**. *Journal of Cancer Survivorship* 2021.

39. Gehring K, Kloek CJJ, Aaronson NK, Janssen KW, Jones LW, Sitskoorn MM, Stuiver MM: **Feasibility of a home-based exercise intervention with remote guidance for patients with stable grade II and III gliomas: a pilot randomized controlled trial**. *Clinical rehabilitation* 2018, **32**(3):352-366.

40. Furzer BJ, Ackland TR, Wallman KE, Petterson AS, Gordon SM, Wright KE, Joske DJL: **A randomised controlled trial comparing the effects of a 12-week supervised exercise versus usual care on outcomes in haematological cancer patients**. *Supportive Care in Cancer* 2016, **24**(4):1697-1707.

41. Shelton ML, Lee JQ, Morris GS, Massey PR, Kendall DG, Munsell MF, Anderson KO, Simmonds MJ, Giralt SA: **A randomized control trial of a supervised versus a self-directed exercise program for allogeneic stem cell transplant patients**. *Psycho-oncology* 2009, **18**(4):353-359.

42. McNeely ML, Parliament MB, Seikaly H, Jha N, Magee DJ, Haykowsky MJ, Courneya KS: **Sustainability of Outcomes after a Randomized Crossover Trial of Resistance Exercise for Shoulder Dysfunction in Survivors of Head and Neck Cancer**. *Physiotherapy Canada* 2015, **67**(1):85-93.

43. Brocki BC, Andreasen J, Nielsen LR, Nekrasas V, Gorst-Rasmussen A, Westerdahl E: **Short and long-term effects of supervised versus unsupervised exercise training on health-related quality of life and functional outcomes following lung cancer surgery - A randomized controlled trial**. *Lung Cancer* 2014, **83**(1):102-108.

44. Broderick JM, Guinan E, Kennedy MJ, Hollywood D, Courneya KS, Culos-Reed SN, Bennett K, DM OD, Hussey J: **Feasibility and efficacy of a supervised exercise intervention in de-conditioned cancer survivors during the early survivorship phase: the PEACH trial**. *J Cancer Surviv* 2013, **7**(4):551-562.

45. Gothe NP, Erlenbach E: **Feasibility of a yoga, aerobic and stretching-toning exercise program for adult cancer survivors: the STAYFit trial**. *Journal of cancer survivorship : research and practice* 2022, **16**(5):1107-1116.

46. Kampshoff CS, Mechelen W, Schep G, Nijziel MR, Witlox L, Bosman L, Chinapaw MJM, Brug J, Buffart LM: **Participation in and adherence to physical exercise after completion of primary cancer treatment**. *International Journal of Behavioral Nutrition and Physical Activity* 2016, **13**(1).

47. Kneis S, Wehrle A, Müller J, Maurer C, Ihorst G, Gollhofer A, Bertz H: **It's never too late - Balance and endurance training improves functional performance, quality of life, and alleviates neuropathic symptoms in cancer survivors suffering from chemotherapy-induced peripheral neuropathy: Results of a randomized controlled trial**. *BMC Cancer* 2019, **19**(1).

48. Lapen K, Benusis L, Pearson S, Search B, Coleton M, Li QS, Sjoberg D, Konner J, Mao JJ, Deng G: **A Feasibility Study of Restorative Yoga Versus Vigorous Yoga Intervention for Sedentary Breast and Ovarian Cancer Survivors**. *International journal of yoga therapy* 2018, **28**(1):79��85.

49. LaStayo PC, Marcus RL, Dibble LE, Smith SB, Beck SL: **Eccentric exercise versus usual-care with older cancer survivors: the impact on muscle and mobility--an exploratory pilot study**. *BMC geriatrics* 2011, **11**:5.

50. Lin PJ, Kleckner IR, Loh KP, Inglis JE, Peppone LJ, Janelsins MC, Kamen CS, Heckler CE, Culakova E, Pigeon WR *et al*: **Influence of Yoga on Cancer-Related Fatigue and on Mediational Relationships Between Changes in Sleep and Cancer-Related Fatigue: A Nationwide, Multicenter Randomized Controlled Trial of Yoga in Cancer Survivors**. *Integrative cancer therapies* 2019, **18**.

51. Martin EA, Battaglini CL, Hands B, Naumann F: **Higher-Intensity Exercise Results in More Sustainable Improvements for VO2peak for Breast and Prostate Cancer Survivors**. *Oncology Nursing Forum* 2015, **42**(3):241-249.

52. Rabin C, Pinto B, Fava J: **Randomized Trial of a Physical Activity and Meditation Intervention for Young Adult Cancer Survivors**. *Journal of Adolescent & Young Adult Oncology* 2016, **5**(1):41-47.

53. Siebert S, Kollikowski A, Minto CA, Byrtus F, Lesnik J, Weis J, Horneber M, Bloch W, Baumann FT, Salchow J: **A Randomized, Controlled Pilot Study to Evaluate the Immediate Effect of Targeted Exercise Therapy on Cancer-Related Fatigue in Cancer Survivors: The FatiGO Study**. *Oncology Research and Treatment* 2022, **45**(11):639-649.

54. Thorsen L, Skovlund E, Strømme SB, Hornslien K, Dahl AA, Fosså SD: **Effectiveness of physical activity on cardiorespiratory fitness and health-related quality of life in young and middle-aged cancer patients shortly after chemotherapy**. *Journal of Clinical Oncology* 2005, **23**(10):2378-2388.

55. Van Weert E, Hoekstra-Weebers J, Grol B, Otter R, Arendzen HJ, Postema K, Sanderman R, Van Der Schans C: **A multidimensional cancer rehabilitation program for cancer survivors: Effectiveness on health-related quality of life**. *Journal of psychosomatic research* 2005, **58**(6):485-496.

56. Van Vulpen JK, Hiensch AE, Van Hillegersberg R, Ruurda JP, Backx FJG, Nieuwenhuijzen GAP, Kouwenhoven EA, Groenendijk RPR, Van Der Peet DL, Hazebroek EJ *et al*: **Supervised exercise after oesophageal cancer surgery: the PERFECT multicentre randomized clinical trial**. *British Journal of Surgery* 2021, **108**(7):786-796.

57. O'Neill LM, Guinan E, Doyle SL, Bennett AE, Murphy C, Elliott JA, O'Sullivan J, Reynolds JV, Hussey J: **The RESTORE Randomized Controlled Trial: Impact of a Multidisciplinary Rehabilitative Program on Cardiorespiratory Fitness in Esophagogastric cancer Survivorship**. *Annals of surgery* 2018, **268**(5):747-755.

58. Zhou Y, Cartmel B, Gottlieb L, Ercolano EA, Li F, Harrigan M, McCorkle R, Ligibel JA, von Gruenigen VE, Gogoi R *et al*: **Randomized Trial of Exercise on Quality of Life in Women With Ovarian Cancer: Women's Activity and Lifestyle Study in Connecticut (WALC)**. *J Natl Cancer Inst* 2017, **109**(12).

59. Alibhai SM, Santa Mina D, Ritvo P, Sabiston C, Krahn M, Tomlinson G, Matthew A, Segal R, Warde P, Durbano S *et al*: **A phase II RCT and economic analysis of three exercise delivery methods in men with prostate cancer on androgen deprivation therapy**. *BMC Cancer* 2015, **15**:312.

60. Bourke L, Doll H, Crank H, Daley A, Rosario D, Saxton JM: **Lifestyle intervention in men with advanced prostate cancer receiving androgen suppression therapy: a feasibility study**. *Cancer Epidemiol Biomarkers Prev* 2011, **20**(4):647-657.

61. Campo RA, Agarwal N, LaStayo PC, O'Connor K, Pappas L, Boucher KM, Gardner J, Smith S, Light KC, Kinney AY: **Levels of fatigue and distress in senior prostate cancer survivors enrolled in a 12-week randomized controlled trial of Qigong**. *J Cancer Surviv* 2014, **8**(1):60-69.

62. Cormie P, Newton RU, Taaffe DR, Spry N, Joseph D, Akhlil Hamid M, Galvão DA: **Exercise maintains sexual activity in men undergoing androgen suppression for prostate cancer: A randomized controlled trial**. *Prostate Cancer and Prostatic Diseases* 2013, **16**(2):170-175.

63. Kim SH, Seong DH, Yoon SM, Choi YD, Choi E, Song Y, Song H: **The Effect on Bone Outcomes of Home-based Exercise Intervention for Prostate Cancer Survivors Receiving Androgen Deprivation Therapy: A Pilot Randomized Controlled Trial**. *Cancer Nursing* 2018, **41**(5):379-388.

64. Livingston PM, Craike MJ, Salmon J, Courneya KS, Gaskin CJ, Fraser SF, Mohebbi M, Broadbent S, Botti M, Kent B: **Effects of a clinician referral and exercise program for men who have completed active treatment for prostate cancer: A multicenter cluster randomized controlled trial (ENGAGE)**. *Cancer* 2015, **121**(15):2646-2654.

65. Nilsen TS, Raastad T, Skovlund E, Courneya KS, Langberg CW, Lilleby W, Fossa SD, Thorsen L: **Effects of strength training on body composition, physical functioning, and quality of life in prostate cancer patients during androgen deprivation therapy**. *Acta oncologica (Stockholm, Sweden)* 2015, **54**(10):1805-1813.

66. Papadopoulos E, Gillen J, Moore D, Au D, Kurgan N, Klentrou P, Finelli A, Alibhai SMH, Santa Mina D: **High-intensity interval training or resistance training versus usual care in men with prostate cancer on active surveillance: a 3-arm feasibility randomized controlled trial**. *Applied physiology, nutrition, and metabolism = Physiologie appliquee, nutrition et metabolisme* 2021, **46**(12):1535-1544.

67. Santa Mina D, Alibhai S MH, Matthew AG, Guglietti CL, Pirbaglou M, Trachtenberg J, Ritvo P: **A randomized trial of aerobic versus resistance exercise in prostate cancer survivors**. *Journal of aging and physical activity* 2013, **21**(4):455-478.
