# Supplementary material for: Retention rates and reasons for non-retention in exercise oncology trials in the post-treatment phase—a systematic review
Source: J Cancer Surviv. 2024 Apr 3;19(5):1535–43. doi: 10.1007/s11764-024-01569-4 (PMC12460456; doi:10.1007/s11764-024-01569-4)
Supplement: Supplementary file 3 — Supplementary file3 (DOCX 53 KB) [file 11764_2024_1569_MOESM3_ESM.docx]

**Supplemental Material 3 – Retention Rates at Subsequent Follow-up Assessments by Cancer Type**

*Title:* *:* Retention Rates and Reasons for Non-Retention in Exercise Oncology Trials in the Post-Treatment Phase- A Systematic Review

*Authors*: Sofia Hu^1^, David Mockler^4^, Emer Guinan^2,3^, Linda O’Neill^2,3^

*Affiliations*:

1. School of Pharmacy and Pharmaceutical Sciences, Trinity College Dublin, University of Dublin, Dublin, Ireland
2. Trinity St. James’s Cancer Institute, Dublin, Ireland.
3. Discipline of Physiotherapy, School of Medicine, Trinity College Dublin, University of Dublin, Dublin, Ireland.
4. John Stearne Library, Trinity Centre for Health Sciences, St. James’s Hospital, Dublin, Ireland.

*Corresponding Author:* Dr Linda O’Neill, Research Fellow, Trinity St James’s Cancer Institute, Dublin 8, Ireland. Email: [loneill4@tcd.ie](mailto:loneill4@tcd.ie)

**Breast**

| **Author (Country)** | **Follow-up at 0-6 months from baseline** | **Follow-up at 7-12 months from baseline** | **Follow-up at 13-18 months from baseline** | **Follow-up at 19-24 months from baseline** | **Follow-up at >24 months from baseline** |
| --- | --- | --- | --- | --- | --- |
| Anderson, 2012 (US) [1] |  | 9 months-  *Overall:* 75  *Intervention:* 82.69  *Control:* 67.51  12 months-  *Overall:* 80.77  *Intervention:* 88.46  *Control:* 73.08 | 15 months- *Overall:* 69.23 *Intervention:* 76.92  *Control:* 61.54  18 months-  *Overall:* 78.85 *Intervention:* 82.09  *Control:* 75 |  |  |
| Cantarero-Villanueva, 2011 (Spain) [2] |  | 8 months-  Overall: 70.5  Intervention: 76.32  Control: 65 |  |  |  |
| Casla, 2015 (Spain) [3] |  | 9 months-  *Overall/Control:* N/A (only intervention group was followed up)  *Intervention:* 76.596 |  |  |  |
| Daley, 2007 (UK) [4] | 6 months-  *Overall:* 88.89  *Intervention:* 91.18  *Control:* Placebo: 94.44, UC: 81.58 |  |  |  |  |
| Dieli-Conwright, 2018 (US) [5] | 3 months-  *Overall/Control:* N/A (only intervention group was followed up)  *Intervention:* 92 |  |  |  |  |
| Greenlee, 2013 (US) [6] |  | 9 months-  *Overall:* 90.48  *Intervention:* 95.45  *Control:* 85  12 months-  *Overall:* 90.48  *Intervention:* 95.45  *Control:* 85 |  |  |  |
| Guinan, 2013 (Ireland) [7] | 5 months-  *Overall:* 84.62  *Intervention:* 87.5  *Control:* 80 |  |  |  |  |
| McNeil, 2019 (Canada) [8] | 6 months-  *Overall:* 91.11 *Intervention:* HI: 93.33 LI: 100 *Control:* 80 |  |  |  |  |
| Milne, 2008 (Australia) [9] | 4.5 months-  *Overall:* 96.55 *Intervention:* 96.55 *Control:* 96.55  6 months-  *Overall:* 98.28 *Intervention:* 96.55 *Control:* 100 |  |  |  |  |
| Sagen, 2009 (Norway) [10] |  |  |  | 24 months-  *Overall:* 70.59  *Intervention:* 57.69  *Control:* 84 |  |

**Colorectal**

| **Author (Country)** | **Follow-up at 0-6 months from baseline** | **Follow-up at 7-12 months from baseline** | **Follow-up at 13-18 months from baseline** | **Follow-up at 19-24 months from baseline** | **Follow-up at >24 months from baseline** |
| --- | --- | --- | --- | --- | --- |
| Pinto, 2013 (US) [11] | 6 months-  *Overall:* 91.3  *Intervention:* 95  *Control:* 88.46 | 12 months-  *Overall:* 92.31  *Intervention:* 95  *Control:* 88.46 |  |  |  |

**Endometrial**

| **Author (Country)** | **Follow-up at 0-6 months from baseline** | **Follow-up at 7-12 months from baseline** | **Follow-up at 13-18 months from baseline** | **Follow-up at 19-24 months from baseline** | **Follow-up at >24 months from baseline** |
| --- | --- | --- | --- | --- | --- |
| Gorzelitz, 2021 (US) [12] | 3.75 months-  *Overall:* 95  *Intervention:* 95  *Control:* 95 |  |  |  |  |

**Haematological**

| **Author (Country)** | **Follow-up at 0-6 months from baseline** | **Follow-up at 7-12 months from baseline** | **Follow-up at 13-18 months from baseline** | **Follow-up at 19-24 months from baseline** | **Follow-up at >24 months from baseline** |
| --- | --- | --- | --- | --- | --- |
| Furzer, 2016 (Australia) [13] | 6 months-  *Overall: 84.09*  *Intervention:* 81.82  *Control:* 86.36 |  |  |  |  |

**Head and Neck**

| **Author (Country)** | **Follow-up at 0-6 months from baseline** | **Follow-up at 7-12 months from baseline** | **Follow-up at 13-18 months from baseline** | **Follow-up at 19-24 months from baseline** | **Follow-up at >24 months from baseline** |
| --- | --- | --- | --- | --- | --- |
| McNeely, 2015 (Canada) [14] |  | 12 months-  *Overall:* 71.15 *Intervention:* 76  *Control:* 66.67 (crossover to the intervention group was allowed) |  |  |  |

**Lung**

| **Author (Country)** | **Follow-up at 0-6 months from baseline** | **Follow-up at 7-12 months from baseline** | **Follow-up at 13-18 months from baseline** | **Follow-up at 19-24 months from baseline** | **Follow-up at >24 months from baseline** |
| --- | --- | --- | --- | --- | --- |
| Brocki, 2014 (Denmark) [15] | 4 months-  *Overall:* 85.90  *Intervention:* 78.05  *Control:* 83.78 | 12 months-  *Overall:* 75.64  *Intervention:* 65.29  *Control:* 83.78 |  |  |  |

**Mixed Population**

| **Author (Country)** | **Follow-up at 0-6 months from baseline** | **Follow-up at 7-12 months from baseline** | **Follow-up at 13-18 months from baseline** | **Follow-up at 19-24 months from baseline** | **Follow-up at >24 months from baseline** |
| --- | --- | --- | --- | --- | --- |
| Broderick, 2013 (Ireland) [16] | 5 months-  *Overall:* 88.37  *Intervention:* 86.96  *Control:* 90 |  |  |  |  |
| Lapen, 2018 (US) [17] | 6 months (End of Unsupervised Intervention)-  *Overall:* 64.29  *Intervention:* 47.62  *Control:* 80.95 |  |  |  |  |
| Martin, 2015 (Australia) [18] | 6.5 months-  *Overall/Control:* N/A (only intervention group was followed up)  *Intervention*: HI: 80 LI 72.72 |  |  |  |  |
| Van Weert, 2005 (Netherlands) [19] | 3 months-  *Overall:* 70.37  *Intervention:* N/R  *Control:* N/R |  |  |  |  |

**Oesophageal**

| **Author (Country)** | **Follow-up at 0-6 months from baseline** | **Follow-up at 7-12 months from baseline** | **Follow-up at 13-18 months from baseline** | **Follow-up at 19-24 months from baseline** | **Follow-up at >24 months from baseline** |
| --- | --- | --- | --- | --- | --- |
| Van Vulpen, 2021 (Netherlands) [20] | 6 months-  *Overall:* 81.67 *Intervention:* 78.69  *Control:* 84.75 |  |  |  |  |

**Oesophagogastric**

| **Author (Country)** | **Follow-up at 0-6 months from baseline** | **Follow-up at 7-12 months from baseline** | **Follow-up at 13-18 months from baseline** | **Follow-up at 19-24 months from baseline** | **Follow-up at >24 months from baseline** |
| --- | --- | --- | --- | --- | --- |
| O’Neill, 2018 (Ireland) [21] | 6 months-  *Overall:* 88.37  *Intervention:* 95.24  *Control:* 81.82 |  |  |  |  |

**Prostate**

| **Author (Country)** | **Follow-up at 0-6 months from baseline** | **Follow-up at 7-12 months from baseline** | **Follow-up at 13-18 months from baseline** | **Follow-up at 19-24 months from baseline** | **Follow-up at >24 months from baseline** |
| --- | --- | --- | --- | --- | --- |
| Bourke, 2011 (UK) [22] | 6 months-  *Overall:* 56  *Intervention:* 60  *Control:* 52 |  |  |  |  |
| SantaMina, 2013 (Canada) [23] |  | 12 months-  *Overall:* 46.97 *Intervention:* 59.375 *Control:* 35.29 |  |  |  |

**References:**

1. Anderson RT, Kimmick GG, McCoy TP, Hopkins J, Levine E, Miller G, Ribisl P, Mihalko SL: **A randomized trial of exercise on well-being and function following breast cancer surgery: The RESTORE trial**. *Journal of Cancer Survivorship* 2012, **6**(2):172-181.

2. Cantarero-Villanueva I, Fernández-Lao C, Díaz-Rodriguez L, Fernández-de-las-Peñas C, Del Moral-Avila R, Arroyo-Morales M: **A multimodal exercise program and multimedia support reduce cancer-related fatigue in breast cancer survivors: A randomised controlled clinical trial**. *European Journal of Integrative Medicine* 2011, **3**(3):e189-e200.

3. Casla S, Lopez-Tarruella S, Jerez Y, Marquez-Rodas I, Galvao DA, Newton RU, Cubedo R, Calvo I, Sampedro J, Barakat R *et al*: **Supervised physical exercise improves VO2max, quality of life, and health in early stage breast cancer patients: a randomized controlled trial**. *Breast Cancer Research & Treatment* 2015, **153**(2):371-382.

4. Daley AJ, Crank H, Saxton JM, Mutrie N, Coleman R, Roalfe A: **Randomized trial of exercise therapy in women treated for breast cancer**. *Journal of Clinical Oncology* 2007, **25**(13):1713-1721.

5. Dieli-Conwright CM, Courneya KS, Demark-Wahnefried W, Sami N, Lee K, Buchanan TA, Spicer DV, Tripathy D, Bernstein L, Mortimer JE: **Effects of aerobic and resistance exercise on metabolic syndrome, sarcopenic obesity, and circulating biomarkers in overweight or obese survivors of breast cancer: A randomized controlled trial**. *Journal of Clinical Oncology* 2018, **36**(9):875-883.

6. Greenlee HA, Crew KD, Mata JM, McKinley PS, Rundle AG, Zhang W, Liao Y, Tsai WY, Hershman DL: **A pilot randomized controlled trial of a commercial diet and exercise weight loss program in minority breast cancer survivors**. *Obesity* 2013, **21**(1):65-76.

7. Guinan E, Hussey J, Broderick JM, Lithander FE, O'Donnell D, Kennedy MJ, Connolly EM: **The effect of aerobic exercise on metabolic and inflammatory markers in breast cancer survivors--a pilot study**. *Supportive care in cancer : official journal of the Multinational Association of Supportive Care in Cancer* 2013, **21**(7):1983-1992.

8. McNeil J, Brenner DR, Stone CR, O'Reilly R, Ruan Y, Vallance JK, Courneya KS, Thorpe KE, Klein DJ, Friedenreich CM: **Activity Tracker to Prescribe Various Exercise Intensities in Breast Cancer Survivors**. *Med Sci Sports Exerc* 2019, **51**(5):930-940.

9. Milne HM, Wallman KE, Gordon S, Courneya KS: **Effects of a combined aerobic and resistance exercise program in breast cancer survivors: a randomized controlled trial**. *Breast Cancer Research & Treatment* 2008, **108**(2):279-288.

10. Sagen Å, Kåresen R, Risberg MA: **Physical activity for the affected limb and arm lymphedema after breast cancer surgery. A prospective, randomized controlled trial with two years follow-up**. *Acta Oncologica* 2009, **48**(8):1102-1110.

11. Pinto BM, Papandonatos GD, Goldstein MG, Marcus BH, Farrell N: **Home-based physical activity intervention for colorectal cancer survivors**. *Psycho-oncology* 2013, **22**(1):54-64.

12. Gorzelitz J, Costanzo E, Gangnon R, Koltyn K, Dietz AT, Spencer RJ, Rash J, Cadmus-Bertram L: **Feasibility and acceptability of home-based strength training in endometrial cancer survivors**. *Journal of Cancer Survivorship* 2021.

13. Furzer BJ, Ackland TR, Wallman KE, Petterson AS, Gordon SM, Wright KE, Joske DJL: **A randomised controlled trial comparing the effects of a 12-week supervised exercise versus usual care on outcomes in haematological cancer patients**. *Supportive Care in Cancer* 2016, **24**(4):1697-1707.

14. McNeely ML, Parliament MB, Seikaly H, Jha N, Magee DJ, Haykowsky MJ, Courneya KS: **Sustainability of Outcomes after a Randomized Crossover Trial of Resistance Exercise for Shoulder Dysfunction in Survivors of Head and Neck Cancer**. *Physiotherapy Canada* 2015, **67**(1):85-93.

15. Brocki BC, Andreasen J, Nielsen LR, Nekrasas V, Gorst-Rasmussen A, Westerdahl E: **Short and long-term effects of supervised versus unsupervised exercise training on health-related quality of life and functional outcomes following lung cancer surgery - A randomized controlled trial**. *Lung Cancer* 2014, **83**(1):102-108.

16. Broderick JM, Guinan E, Kennedy MJ, Hollywood D, Courneya KS, Culos-Reed SN, Bennett K, DM OD, Hussey J: **Feasibility and efficacy of a supervised exercise intervention in de-conditioned cancer survivors during the early survivorship phase: the PEACH trial**. *J Cancer Surviv* 2013, **7**(4):551-562.

17. Lapen K, Benusis L, Pearson S, Search B, Coleton M, Li QS, Sjoberg D, Konner J, Mao JJ, Deng G: **A Feasibility Study of Restorative Yoga Versus Vigorous Yoga Intervention for Sedentary Breast and Ovarian Cancer Survivors**. *International journal of yoga therapy* 2018, **28**(1):79��85.

18. Martin EA, Battaglini CL, Hands B, Naumann F: **Higher-Intensity Exercise Results in More Sustainable Improvements for VO2peak for Breast and Prostate Cancer Survivors**. *Oncology Nursing Forum* 2015, **42**(3):241-249.

19. Van Weert E, Hoekstra-Weebers J, Grol B, Otter R, Arendzen HJ, Postema K, Sanderman R, Van Der Schans C: **A multidimensional cancer rehabilitation program for cancer survivors: Effectiveness on health-related quality of life**. *Journal of psychosomatic research* 2005, **58**(6):485-496.

20. Van Vulpen JK, Hiensch AE, Van Hillegersberg R, Ruurda JP, Backx FJG, Nieuwenhuijzen GAP, Kouwenhoven EA, Groenendijk RPR, Van Der Peet DL, Hazebroek EJ *et al*: **Supervised exercise after oesophageal cancer surgery: the PERFECT multicentre randomized clinical trial**. *British Journal of Surgery* 2021, **108**(7):786-796.

21. O'Neill LM, Guinan E, Doyle SL, Bennett AE, Murphy C, Elliott JA, O'Sullivan J, Reynolds JV, Hussey J: **The RESTORE Randomized Controlled Trial: Impact of a Multidisciplinary Rehabilitative Program on Cardiorespiratory Fitness in Esophagogastric cancer Survivorship**. *Annals of surgery* 2018, **268**(5):747-755.

22. Bourke L, Doll H, Crank H, Daley A, Rosario D, Saxton JM: **Lifestyle intervention in men with advanced prostate cancer receiving androgen suppression therapy: a feasibility study**. *Cancer Epidemiol Biomarkers Prev* 2011, **20**(4):647-657.

23. Santa Mina D, Alibhai S MH, Matthew AG, Guglietti CL, Pirbaglou M, Trachtenberg J, Ritvo P: **A randomized trial of aerobic versus resistance exercise in prostate cancer survivors**. *Journal of aging and physical activity* 2013, **21**(4):455-478.
